# Intraspecies Signaling between Common Variants of Pseudomonas aeruginosa Increases Production of Quorum-Sensing-Controlled Virulence Factors

**DOI:** 10.1128/mBio.01865-20

**Published:** 2020-08-25

**Authors:** Dallas L. Mould, Nico J. Botelho, Deborah A. Hogan

**Affiliations:** a Geisel School of Medicine at Dartmouth, Hanover, New Hampshire, USA; Emory University School of Medicine

**Keywords:** *Pseudomonas aeruginosa*, RhlR, citrate, intraspecies interactions, *lasR*, pyochelin, quorum sensing

## Abstract

The opportunistic pathogen Pseudomonas aeruginosa damages hosts through the production of diverse secreted products, many of which are regulated by quorum sensing (QS). The *lasR* gene, which encodes a central QS regulator, is frequently mutated in clinical isolates from chronic infections, and loss of LasR function (LasR−) generally impairs the activity of downstream QS regulators RhlR and PqsR. We found that in cocultures containing LasR+ and LasR− strains, LasR− strains hyperproduce the RhlR/RhlI-regulated antagonistic factors pyocyanin and rhamnolipids in diverse models and media and in different strain backgrounds. Diffusible QS autoinducers produced by the wild type were not required for this effect. Using transcriptomics, genetics, and biochemical approaches, we uncovered a reciprocal interaction between wild-type and *lasR* mutant pairs wherein the iron-scavenging siderophore pyochelin produced by the *lasR* mutant induced citrate release and cross-feeding from the wild type. Citrate, a metabolite often secreted in low iron environments, stimulated RhlR signaling and RhlI levels in LasR−but not in LasR+ strains. These studies reveal the potential for complex interactions between recently diverged, genetically distinct isolates within populations from single chronic infections.

## Abstract (precis)

Coculture interactions between *lasR* loss-of-function and LasR+ Pseudomonas aeruginosa strains may explain the worse outcomes associated with the presence of LasR− strains. More broadly, this report illustrates how interactions within a genotypically diverse population, similar to those that frequently develop in natural settings, can promote unpredictably high virulence factor production.

## INTRODUCTION

In chronic infections and healthy microbiomes, genetic diversity frequently arises and persists within clonally derived microbial populations, and recent data highlight that heterogeneity within a population can pose challenges to clearance and treatment (1–4). Genotypic and phenotypic complexity is particularly well documented in the chronic lung infections associated with the genetic disease cystic fibrosis (CF), and studies have convincingly demonstrated that within a species, a common set of genes is under selection across strains and hosts (5–11).

Loss-of-function mutations in Pseudomonas aeruginosa *lasR* (LasR−) are commonly found in CF isolates and strains from acute infections and from environmental sources (12–16). LasR participates in the regulation of quorum sensing (QS) in conjunction with other transcription factors, including RhlR and PqsR (MvfR). These regulators have one or more autoinducer ligands: 3-oxo-C_12_-homoserine lactone (3OC12HSL) for LasR, C_4_-homoserine lactone (C4HSL) for RhlR, and hydroxy-alkyl-quinolones (pseudomonas quinolone signal [PQS] and hydroxy-heptyl quinolone [HHQ]) for PqsR (17). In the regulatory networks described in widely used P. aeruginosa model strains, LasR is an upstream regulator of RhlR and PqsR signaling, and together these regulators control the expression of a suite of genes associated with virulence, including redox-active small-molecule phenazines (18–20), cyanide (21), and rhamnolipid surfactants important for surface motility, biofilm dispersal, and host cell damage (22–24).

Although LasR positively regulates virulence factors, and *lasR* loss-of-function mutants have reduced virulence in infection models, LasR− strain culture positivity is correlated with worse disease outcomes in acute and chronic infections (12, 13). There are several possible explanations for this apparent contradiction. LasR− clinical isolates (CIs) are frequently found among strains with functional LasR (LasR+) where exoproducts can be shared or signal cross-feeding can occur (14), and some LasR− clinical isolates exhibit rewired QS regulation (25). Loss of LasR function also confers some fitness advantages, including altered catabolic profiles (26) and enhanced growth in low oxygen (27, 28), which may contribute to bacterial burden. Further, LasR− strains can activate QS in response to specific fungal products (29) or culture conditions (30, 31).

In addition to LasR status, iron acquisition strategies are often heterogeneous across P. aeruginosa isolates. P. aeruginosa procures iron through the use of siderophores, including pyochelin (32–34) and pyoverdine (35), from heme, or through a direct iron uptake system (36–38). Although it is common to encounter P. aeruginosa strains with loss-of-function mutations in genes required for biosynthesis of the high-affinity siderophore pyoverdine, genes associated with use of pyochelin, heme utilization, and ferrous iron import are generally intact (39–41). Iron limitation can deprioritize pathways that require abundant iron including the tricarboxylic acid (TCA) cycle (42), and consequently *Pseudomonas* spp. and other organisms release metabolic intermediates, such as citrate, that accumulate at iron-requiring steps (e.g., aconitase) (43, 44).

Here, we show that mixtures of P. aeruginosa LasR− and LasR+ strains had enhanced production of QS-controlled factors across media, culture conditions, and strain backgrounds. The unpredictably high levels of exoproducts in coculture were produced by LasR− strains due to activation of RhlR, likely through increased C4HSL synthase (RhlI) stability in LasR− strains. Our genetic, transcriptomic, and biochemical studies led us to uncover a set of interactions in which production of the siderophore pyochelin by ∆*lasR* cells induced citrate release by wild type (WT) but not by ∆*lasR* cells. We found that citrate led to increased RhlI protein levels and RhlR activity in ∆*lasR* cells but not in the WT. Together, these intraspecies interactions increased production of exoproducts known to cause host damage.

## RESULTS

### P. aeruginosa ∆*lasR* overproduces pyocyanin in coculture with the wild type.

**FIG 1:**
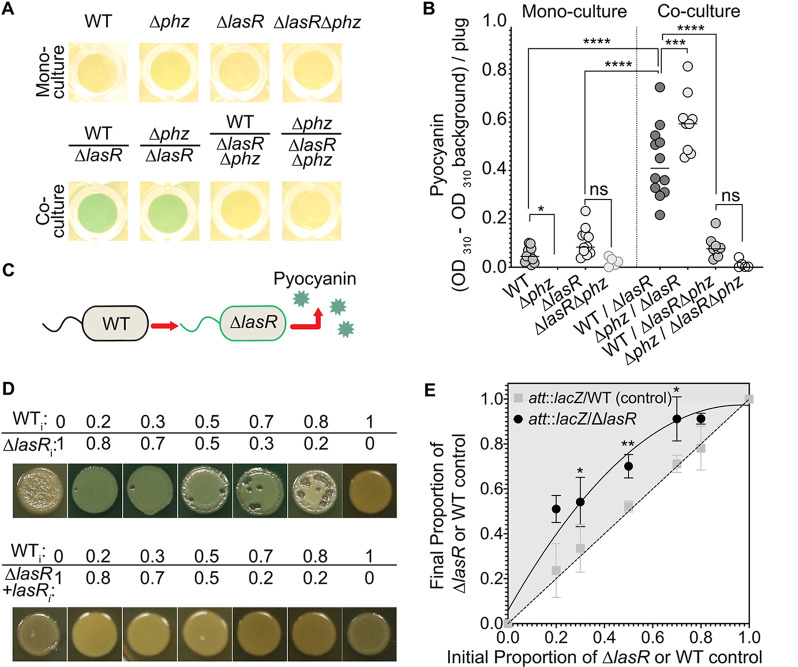
The ∆*lasR* strain produces pyocyanin in wild-type/∆*lasR* cocultures beyond monoculture concentrations. (A) Representative images of the wild-type (WT) and ∆*lasR* strains and their phenazine-deficient derivatives (∆*phz* strains) visualized from the bottom of a 96-well LB agar plate after 16 h growth as mono- and cocultures with 70:30 WT-to-∆*lasR* cell initial ratio. (B) Pyocyanin levels above background, defined as the average signal for the ∆*lasR* ∆*phz* strain, quantified for cultures described in panel A. ns, not significant; *, *P* < 0.05; ***, *P* < 0.0005; ****, *P* < 0.0001, as determined by ordinary one-way analysis of variance with Tukey’s multiple-comparison test for *n* ≥ 8 replicates on three different days. (C) Model of pyocyanin production by the ∆*lasR* strain in coculture with the WT. (D) Representative pyocyanin production of the wild type cocultured with the ∆*lasR* strain or the ∆*lasR* strain complemented with the *lasR* gene at the native locus (∆*lasR* + *lasR* strain) across several initial (designated by the subscript i) proportions on LB medium for 20 h. Three biological replicates were included in at least 3 independent experiments. (E) Average final proportion of 3 replicate colony biofilms quantified after 16 h growth for the WT strain and the ∆*lasR* strain cocultured with a WT strain tagged with *lacZ* in 3 independent experiments. Experimental setup was as described in panel D. *, *P* < 0.05; **, *P* < 0.005, as determined by two-tailed *t* tests of paired ratios between *att*::*lacZ*/WT (control) and *att*::*lacZ*/∆*lasR* cocultures at each initial ratio. All results that reach significance are marked.

We observed that mixtures of P. aeruginosa LasR+ and LasR− strains had high levels of total pyocyanin, a secreted, blue-pigmented phenazine. As shown in spot colonies of the PA14 wild-type (WT), ∆*lasR* strain, and WT and ∆*lasR* strain cocultures (here, WT/∆*lasR* cocultures), the strain mixture showed increased blue pigmentation (Fig. 1A) and a significant 4-fold induction of pyocyanin above the background relative to the level with either strain alone (Fig. 1B). Phenazine-deficient derivatives, ∆*phz* (∆*phzA1* ∆*phzB1* ∆*phzC1* ∆*phzD1* ∆*phzE1* ∆*phzF1* ∆*phzG1* ∆*phzA2* ∆*phzB2* ∆*phzC2* ∆*phzD2* ∆*phzE2* ∆*phzF2* ∆*phzG2*) and ∆*lasR* ∆*phz* (∆*lasR* ∆*phzC1* ∆*phzC2*) strains, were also included, and as expected, ∆*phz* and ∆*lasR* ∆*phz* cells showed no blue colony pigmentation (Fig. 1A). The higher levels of pyocyanin in WT/∆*lasR* cocultures relative to levels in single-strain cultures were also observed on artificial sputum medium (ASM) and on phosphate-buffered medium with or without amino acids, indicating that the phenomenon occurred under diverse conditions (see Fig. S1A in the supplemental material). Cocultures of clonally derived LasR+ and LasR− clinical isolates collected from single respiratory sputum samples from chronically infected individuals with cystic fibrosis (14) also had increased production of pyocyanin relative to monoculture levels when LasR+ and LasR− strains were grown together (Fig. S1B and C).

10.1128/mBio.01865-20.1 FIG S1 Increased pyocyanin production of LasR+ and LasR− strain cocultures relative to either strain alone is stable across media and strains. (A) Representative images from PA14 wild type (WT) and ∆*lasR* strain mono- and coculture pyocyanin production on rich medium (LB and artificial sputum medium [ASM]), and minimal (M63 medium, pH 6.8, 0.2% glucose; no Casamino Acids [CAA]) and defined phosphate buffered medium (M63 medium, pH 6.8, 0.2% glucose + 0.2% CAA). (B) Pyocyanin production of mono- and cocultures of strain PA14 WT and the ∆*lasR* strain and clonally derived clinical isolates DH2417 (LasR+) and DH2415 (LasR−). (C) Pyocyanin quantification of LasR+ and LasR− strains grown in mono- and coculture on 96-well LB agar plugs for 16 h. Strain PA14 WT and the ∆*lasR* strain and previously characterized clinical isolate pairs DH2417 (LasR+) and DH2415 (LasR−) and DH1133 (LasR+) and DH1132 (LasR−), from two distinct CF patients, are also shown. Data are from two independent experiments with four biological replicates each. (D) Number of CFU in 16-h colony biofilms grown on LB medium. Each strain set is presented relative to the level of the WT or the LasR+ strain. PA14 WT and ∆*lasR* strain mono- and coculture data are the average from four independent experiments with at least three biological replicates. ns, not significant; **, *P* < 0.005; ***, *P* < 0.0005, as determined by analysis of variance with Tukey multiple hypotheses correction for clinical isolate CFU counts. Download FIG S1, PDF file, 0.3 MB. Copyright © 2020 Mould et al. 2020 Mould et al. This content is distributed under the terms of the Creative Commons Attribution 4.0 International license.

To assess individual strain contributions to increased pyocyanin in WT/∆*lasR* cocultures, we replaced each strain with its phenazine-deficient derivative and measured pyocyanin in coculture. When the ∆*lasR* strain was cultured with the phenazine biosynthesis mutant ∆*phz* strain (∆*phz*/∆*lasR* coculture), we still observed increased blue pigmentation and total pyocyanin at a level above that of either monoculture (Fig. 1A and B). Surprisingly, pyocyanin production by ∆*phz*/∆*lasR* cocultures was statistically higher than that of WT/∆*lasR* coculture (Fig. 1B). In contrast, WT/∆*lasR* ∆*phz* cocultures did not display the high-pyocyanin phenotype (Fig. 1A and B) and resembled ∆*phz*/∆*lasR* ∆*phz* cocultures, where no pyocyanin was produced. Collectively, these data suggested that WT/∆*lasR* cocultures produced more pyocyanin than either monoculture alone and that the ∆*lasR* strain contributed the pyocyanin in coculture (Fig. 1C).

That the levels of pyocyanin in WT/∆*lasR* cocultures were higher than the level in each strain alone was not dependent on the initial ratios of WT to ∆*lasR* cells (Fig. 1D). We saw increased coculture colony pigmentation when the initial proportions of WT cells were at 0.2, 0.3, 0.5, 0.7, and 0.8 of the initial inoculums, with the balance comprised of ∆*lasR* cells (Fig. 1D).

No increase in pyocyanin was observed at any ratio when the WT was cocultured with the ∆*lasR* complemented derivative strain (∆*lasR* + *lasR*), indicating that the phenomenon was dependent on the *lasR* mutation (Fig. 1D). To assess the relative abundances of WT and ∆*lasR* cells in coculture, we competed each strain against a neutrally tagged WT strain (PA14 *att*::*lacZ*). We found that ∆*lasR* cells increased in proportion after 16 h of growth in colony biofilms regardless of the starting proportion whereas the proportions of untagged WT cells remained stable (Fig. 1E). We have previously shown that Anr activity is higher in ∆*lasR* strains and contributes to the competitive fitness of the ∆*lasR* strain against WT P. aeruginosa in colony biofilms (27, 45), but Anr was not required for coculture pyocyanin production (Fig. S2).

10.1128/mBio.01865-20.2 FIG S2 ∆*lasR* strain shows Anr-independent increases in pyocyanin in coculture with LasR+ P. aeruginosa. Representative images of WT, ∆*anr*, and ∆*lasR* ∆*anr* strains in mono- and coculture colony biofilms on LB medium after 24 h. Download FIG S2, PDF file, 0.1 MB. Copyright © 2020 Mould et al. 2020 Mould et al. This content is distributed under the terms of the Creative Commons Attribution 4.0 International license.

Pyocyanin is a product regulated by quorum sensing (QS) through the transcription factors LasR, RhlR, and PqsR (46–48), and because QS regulation is cell density dependent, it was important to assess the coculture population size relative to that of the monoculture. Total CFU counts did not increase in WT/∆*lasR* mixed cultures relative to the level for either strain alone (Fig. S1D). Instead, we found that WT/∆*lasR* cocultures had fewer CFU than WT monocultures on lysogeny broth (LB) medium (Fig. S1D). Taken together, these data suggested that altered behavior, rather than cell number, contributed to the increased phenazine profile of LasR− strains.

### Independent of its ability to produce autoinducers, the WT promotes RhlR/I-dependent signaling in a ∆*lasR* strain.

**FIG 2:**
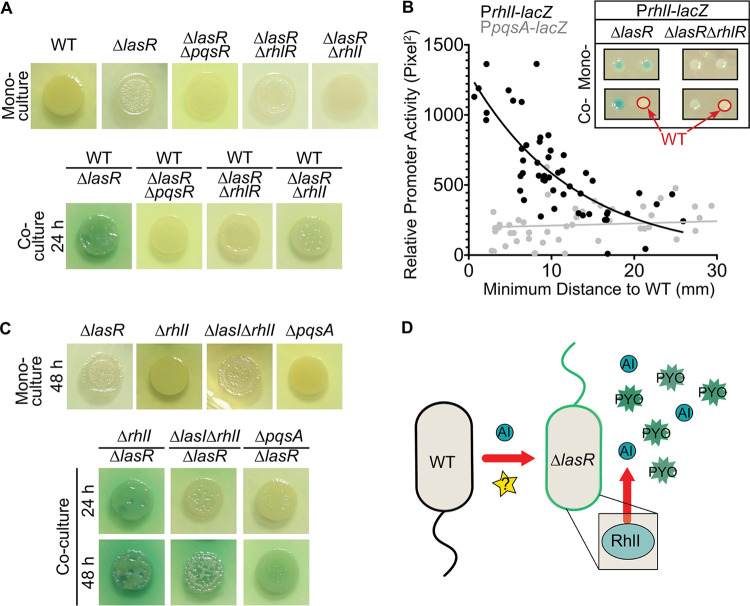
P. aeruginosa WT induces RhlR/I-dependent pyocyanin production in ∆*lasR* cells even in the absence of WT autoinducers. (A) Representative pyocyanin production by monocultures and WT cocultures (70% WT at time 0) of ∆*lasR* and ∆*lasR* strain derivatives that are deficient in PQS or RhlR/I-dependent quorum sensing on LB medium after 24 h growth. (B) Promoter activity of the ∆*lasR* P*pqsA*-*lacZ* (gray) and ∆*lasR* P*rhlI*-*lacZ* (black) strains, quantified by relative pixel intensity of single-cell-derived ∆*lasR* colonies grown near unmodified WT colonies. Solid best-fit nonlinear lines are for visualization. The inset shows representative colonies for RhlR-dependent ∆*lasR* P*rhlI*-*lacZ* strain activity in monoculture and coculture with the WT (red circles). The experiment was repeated with 2 replicates on at least 3 independent days. (C) Representative monoculture and ∆*lasR* coculture images for the ∆*pqsA*, ∆*rhlI*, and ∆*lasI* ∆*rhlI* mutants on LB medium after 24 h and 48 h. (30% ∆*lasR* cells at time 0). (D) Model of AHL-dependent and -independent induction of RhlR/I-dependent activity and phenazine production in ∆*lasR* cells grown in coculture with the WT.

In the canonical QS pathway, LasR regulates both PqsR and RhlR, and mutants lacking either regulator in a WT background have impaired pyocyanin production (49, 50). Both *pqsR* and *rhlR* were required in ∆*lasR* cells for pyocyanin production in coculture with the WT (Fig. 2A). To determine if coculture increased RhlR- or PqsR-dependent signaling in ∆*lasR* strains, we fused *lacZ* to the promoters of *rhlI* and *pqsA* (P*rhlI* and P*pqsA*, respectively) which provide activity readouts of each respective regulator (17). We examined the interactions between WT and the ∆*lasR* strain in single-cell-derived colonies by spreading suspensions containing ∼50 cells of WT with ∼50 cells of either a ∆*lasR* P*rhlI*-*lacZ* or ∆*lasR* P*pqsA*-*lacZ* strain on LB agar containing the colorimetric β-galactosidase substrate 5-bromo-4-chloro-3-indolyl-d-galactopyranoside (X-Gal). Intercolony distances and β-galactosidase activity in ∆*lasR* strains were measured. We found that the rise in P*rhlI*-*lacZ* activity was inversely correlated with the distance to a WT colony (Fig. 2B). Pearson correlation analyses showed that 54% of the variability in ∆*lasR* P*rhlI*- *lacZ* strain activity could be explained by changes in the distance to a WT colony (*P* value of ≤ 0.0001). The increased P*rhlI*-*lacZ* activity in the ∆*lasR* strain was not observed in the ∆*lasR* ∆*rhlR* strain, and close proximity to another ∆*lasR* P*rhlI*-*lacZ* colony did not affect promoter activity (Fig. 2B, inset). Because C4HSL (which is synthesized by RhlI) activates RhlR and because proximity to the WT stimulated ∆*lasR* P*rhlI*-*lacZ* strain activity, we examined the role of RhlI in the ∆*lasR* strain response. We observed that a ∆*lasR* ∆*rhlI* strain was greatly impaired in the induction of pyocyanin upon coculture with the WT (Fig. 2A), which suggests that WT production of C4HSL was insufficient to complement the ∆*lasR* ∆*rhlI* strain and further posits activation of RhlR and C4HSL synthesis in ∆*lasR* strains. Although PqsR was required in ∆*lasR* cells for coculture pyocyanin production, there was no significant correlation with proximity to the WT for ∆*lasR* P*pqsA*-*lacZ* strain activity (Fig. 2B). Collectively, these data indicated that a diffusible factor produced by the WT stimulated RhlR-dependent signaling in the ∆*lasR* strain to induce downstream production of RhlR- and PqsR-dependent factors.

Given differences in colony pigmentation between WT/∆*lasR* ∆*rhlR* and WT/∆*lasR* ∆*rhlI* (Fig. 2A) cocultures, C4HSL cross-feeding between the WT and ∆*lasR* strain likely occurred. Because C4HSL is diffusible and produced by WT cells, we tested the hypothesis that C4HSL or other acyl-homoserine lactones (AHLs) produced by the WT were necessary to induce RhlR-dependent activity in ∆*lasR* cells cocultured with the WT. To test this hypothesis, we cocultured the ∆*lasR* strain with ∆*rhlI* cells or ∆*lasI* ∆*rhlI* cells which lack both acyl-homoserine lactone synthases. Surprisingly, we found that like WT/∆*lasR* cocultures, ∆*rhlI*/∆*lasR* cocultures had higher levels of pyocyanin than monocultures (Fig. 2C). Similarly, ∆*lasI* ∆*rhlI*/∆*lasR* cocultures had higher levels of pyocyanin production than monocultures though the interaction was delayed by ∼24 h relative to the interaction of the WT/∆*lasR* cocultures (Fig. 2C). Consistent with the activity of the ∆*lasR* P*pqsA*-*lacZ* strain, which was not induced in coculture with WT, the PQS-deficient ∆*pqsA* strain supported high pyocyanin colony pigmentation in coculture with ∆*lasR* cells after 24 h of extended incubation (Fig. 2C). The AHL-independent activation observed in ∆*lasI* ∆*rhlI*/∆*lasR* cocultures and the striking differences in pyocyanin production observed between the strongly stimulating ∆*rhlI*/∆*lasR* cocultures and the weakly stimulating WT/∆*lasR* ∆*rhlI* cocultures suggested that the ∆*lasR* strain may rely more heavily on production of its own autoinducer for activation in coculture. Consistent with this model, we found that the ∆*lasR* strain produces RhlR/RhlI (RhlR/I)-dependent AHLs in coculture with an AHL-sensing reporter strain (i.e., ∆*lasI* ∆*rhlI* strain with a *lacZ* promoter fusion to an AHL-responsive gene) (Fig. S3A and B). The dispensable contribution of WT-produced autoinducers implicated a novel signaling interaction in coculture-dependent activation of RhlR/I activity in the ∆*lasR* strain (Fig. 2D).

10.1128/mBio.01865-20.3 FIG S3 The ∆*lasR* strain produced RhlR/I-dependent acyl-homoserine lactone (AHL) autoinducers in coculture with the ∆*lasI ∆rhlI* strain, and this was repressed by iron supplementation. (A) Schematic of experimental setup and quantification of AHL activity for colony biofilms grown on an AHL-sensing reporter strain (i.e., ∆*lasI ∆rhlI* strain with a *lacZ* promoter fusion to an AHL-responsive gene). (B) AHL activity for the WT, AHL-deficient ∆*lasI ∆rhlI* control, ∆*lasR, ∆lasR* ∆*rhlR*, and ∆*lasR ∆rhlI* strains grown on an AHL-sensing reporter strain in X-Gal-containing medium. (C) Representative image of RhlI-dependent activity (i.e., C4HSL) for the ∆*lasR* strain grown on an AHL-sensing reporter strain across an iron gradient under stable X-Gal-containing conditions. (D) Setup and quantification as described for panel a for the ∆*lasR* strain on LB medium (−) and on LB medium plus 10 μM FeSO_4_ (+) including a C4HSL-deficient derivative ∆*lasR ∆rhlI* strain. Data points are from ≥ 3 different days. *, *P* < 0.05; ****, *P* < 0.0001, as determined by ordinary one-way analysis of variance with Dunnett’s multiple-comparison test to the control ∆*lasR* strain. Download FIG S3, PDF file, 2.4 MB. Copyright © 2020 Mould et al. 2020 Mould et al. This content is distributed under the terms of the Creative Commons Attribution 4.0 International license.

To assess whether WT-induced RhlR activity in the ∆*lasR* strain was sufficient to elicit other RhlR/I-controlled phenotypes in addition to pyocyanin production, we tested whether coculture with LasR+ strains enhanced swarming, a surface-associated motility which requires the production of RhlR-regulated rhamnolipid surfactants (51). While the rhamnolipid-defective mutant ∆*rhlA*, ∆*lasR*, and ∆*lasR* ∆*rhlR* strains were unable to swarm, cocultures of the ∆*lasR* strain with the ∆*rhlA* strain swarmed considerably. The phenomenon was dependent on RhlR as the ∆*rhlA*/∆*lasR* ∆*rhlR* cocultures did not swarm (Fig. S4). Altogether, these data implicated broad activation of RhlR-mediated QS in LasR− strains cocultured with LasR+ P. aeruginosa.

10.1128/mBio.01865-20.4 FIG S4 The ∆*lasR* strain showed enhanced production of RhlR-regulated rhamnolipid surfactant in coculture with the LasR+ strain. RhlR-regulated swarming motility on soft agar of the WT, rhamnolipid surfactant biosynthesis mutant (∆*rhlA* strain), ∆*lasR*, and ∆*lasR* ∆*rhlR* strains in mono- and coculture after 36 h. Download FIG S4, PDF file, 0.3 MB. Copyright © 2020 Mould et al. 2020 Mould et al. This content is distributed under the terms of the Creative Commons Attribution 4.0 International license.

### Pyochelin production by ∆*lasR* cells is required for coculture interactions.

**FIG 3:**
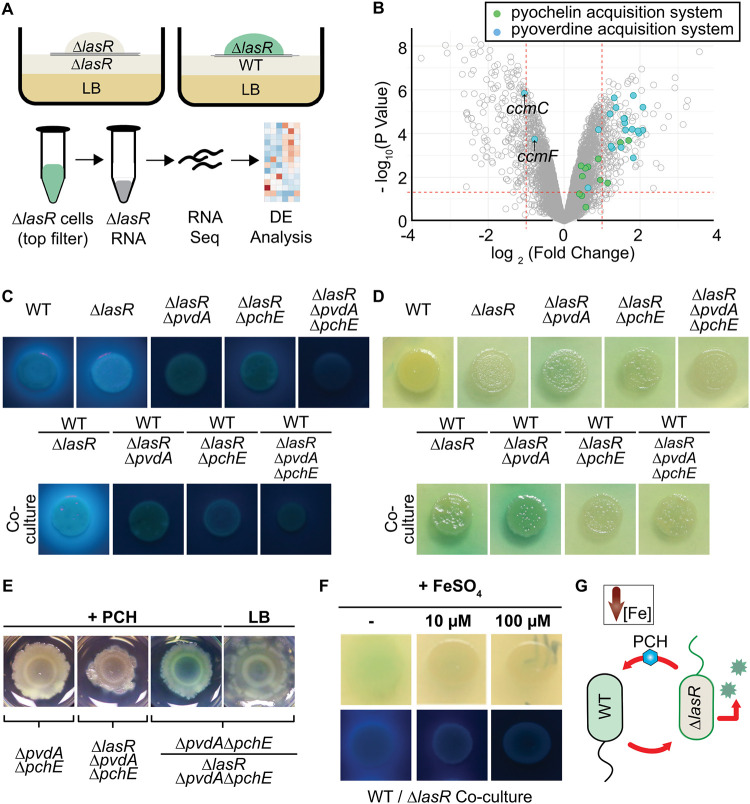
Biosynthesis of the coculture-induced iron scavenging siderophore pyochelin is required in the ∆*lasR* strain for pyocyanin production when it was cultured with the wild type (WT). (A) Scheme for the collection of RNA from ∆*lasR* colony biofilms grown above a lawn of ∆*lasR* or WT cells. DE, differential expression. (B) Volcano plot showing differential expression (log_2_) for ∆*lasR* cells grown over WT relative to ∆*lasR* cells grown over ∆*lasR* on the *x* axis; the *y* axis shows the −log_10_ *P* value for the difference between sample types. Genes involved in pyoverdine (blue) and pyochelin (green) iron acquisition systems are indicated. *ccmC* and *ccmF* (indicated with arrows) of the pyoverdine GO term are involved in *c*-type cytochrome biosynthesis, and strains with knockouts of these genes are reported to produce more pyochelin. (C) Monocultures and WT cocultured with ∆*lasR* strains deficient in pyoverdine (∆*pvdA*) and/or pyochelin (∆*pchE*) biosynthesis. Colonies were visualized under UV light in order to see fluorescent siderophores. Images are representative of at least 3 independent experiments. (D) Pyocyanin production visualized for the colonies shown in panel C. (E) Representative pyocyanin production by siderophore-deficient strains grown in mono- and coculture on LB medium or on LB medium with pyochelin-containing extract (+PCH). Colonies were grown in a 12-well plate with a 2-ml total volume and imaged after 24 h. (F) Mixed colony biofilms of wild-type and ∆*lasR* strains grown on LB medium (−) or LB medium supplemented with either 10 or 100 μM FeSO_4_ visualized under ambient (top) and UV (bottom) light. (G) Model showing that pyochelin (PCH) production by the ∆*lasR* strain is required for WT/∆*lasR* coculture phenazines.

With evidence indicating that induction of RhlR activity in ∆*lasR* cells can occur in both mixed-strain spot colonies and adjacent colonies independent of autoinducer cross-feeding, we sought to gain further insight into the mechanisms that underlie the WT-∆*lasR* cell interactions. We investigated the transcriptomes of the *lasR* mutant in coculture with either the WT or itself via RNA sequencing (RNA-seq). We grew ∆*lasR* colony biofilms on LB medium physically separated from a lawn of either the ∆*lasR* or WT strain by two filters with 0.22-μm pores to prevent mixing of genotypes while allowing for the passage of small molecules. In order to examine ∆*lasR* strain transcriptional profiles, RNA was extracted from cells within the ∆*lasR* colony biofilms grown on the topmost filter for 16 h (Fig. 3A). As expected, no *lasR* reads were detected in our sequencing data to suggest that the wild type was sufficiently excluded by filter separation. Expression levels of a total of 199 genes in the ∆*lasR* strain were higher, and those of 198 genes were lower by a |log_2_(fold change)| of ≥1 with a *P* value of <0.05, in coculture with the WT than levels in the ∆*lasR* strain alone (Table S1). Gene Ontology (GO) term analyses through PantherDB (52) indicated that the upregulated gene set was significantly overrepresented in two pathways related to siderophore biosynthesis: the pyoverdine biosynthetic process and salicylic acid biosynthetic process (an upstream precursor of pyochelin) with ∼44- and ∼77-fold enrichment, respectively (*P* values of <0.005). Twenty-eight out of the 33 genes in the pyochelin and pyoverdine siderophore biosynthesis- and acquisition-related GO families were significantly upregulated in ∆*lasR* cells upon coculture with the WT (Fig. 3B) (i.e.,|log_2_(fold change)| of ≥0 with a *P* value of <0.05). Other low-iron-responsive genes were differentially expressed, including the *has* genes involved in heme uptake and *antABC* genes (Table S1). While we observed stimulation of *rhlI* promoter activity and increased production of RhlR-regulated products, we did not see a broad transcriptional pattern indicative of RhlR activation at this early time point (Table S1), and this point is discussed below.

10.1128/mBio.01865-20.8 TABLE S1 Differentially expressed genes in the ∆*lasR* strain grown with the wild type versus itself in filter-separated cocultures. PA14 number or gene name, if available, is listed in descending order by log_2_(∆*lasR* cells grown on WT/∆*lasR* cells grown on ∆*lasR* cells). Differential expression is defined as |log_2_(∆*lasR* cells grown on WT/∆*lasR* cells grown on ∆*lasR* cells)| >1 with a *P* value of <0.05. Download Table S1, XLSX file, 0.02 MB. Copyright © 2020 Mould et al. 2020 Mould et al. This content is distributed under the terms of the Creative Commons Attribution 4.0 International license.

Given that siderophore biosynthesis genes were upregulated in ∆*lasR* cells cocultured with WT cells, we qualitatively examined production of fluorescent pyoverdine and pyochelin siderophores in monocultures and cocultures. To determine the contribution of both pyoverdine and pyochelin by the ∆*lasR* strain to fluorescence, genes required for pyoverdine biosynthesis (∆*lasR* ∆*pvdA* strain), pyochelin biosynthesis (∆*lasR* ∆*pchE* strain), or both pathways (∆*lasR* ∆*pvdA* ∆*pchE* strain) were disrupted (Fig. 3C). Increased fluorescence attributable to both pyoverdine and pyochelin in coculture was due to siderophore production by ∆*lasR* strains, consistent with the RNA-seq data, as the increased fluorescence in WT/∆*lasR* cocultures was lost in coculture when the ∆*lasR* strain was replaced with a ∆*lasR* ∆*pvdA*, ∆*lasR* ∆*pchE*, or ∆*lasR* ∆*pvdA* ∆*pchE* mutant. While cocultures of the WT and the pyoverdine-deficient derivative ∆*lasR* ∆*pvdA* strain (i.e., WT/∆*lasR* ∆*pvdA* coculture) showed increased pyocyanin production relative to that of either monoculture, the ∆*lasR* ∆*pchE* and ∆*lasR* ∆*pvdA* ∆*pchE* strains did not promote pyocyanin production in coculture with the WT, as observed by colony pigmentation (Fig. 3D). The decrease in ∆*lasR* strain-derived pyocyanin was not due to decreased fitness as disruption of *pvdA* and *pchE* individually in ∆*lasR* cells had no effect on the final proportions; in contrast, the ∆*lasR* ∆*pvdA* ∆*pchE* strain had a significant defect in competitive fitness compared to the fitness of the ∆*lasR* parental strain (Fig. S5). These data suggested that pyochelin played a role in the coculture interaction. To test this model, we complemented the pyocyanin defect in the siderophore-deficient ∆*pvdA ∆pchE*/∆*lasR ∆pchE* coculture with pyochelin-containing extracts from cultures of ∆*pvdA* cells which cannot produce pyoverdine or control extracts from siderophore-deficient ∆*pvdA* ∆*pchE* cultures (Fig. S6A gives supernatant absorption spectra). The two extracts were analyzed using a chrome azurol S (CAS) assay (53) to confirm that chelator activity was present in the ∆*pvdA* cell supernatant extracts but not in extracts from ∆*pvdA* ∆*pchE* cultures (Fig. S6B). Medium supplemented with pyochelin-containing extracts, but not siderophore-free extracts, restored pyocyanin production in ∆*pvdA* ∆*pchE*/∆*lasR* ∆*pchE* cocultures (Fig. 3E), lending further support to the idea that pyochelin was required for coculture interactions. Consistent with this requirement, iron supplementation suppressed siderophore production, as expected, and diminished coculture pyocyanin in WT/∆*lasR* cocultures (Fig. 3F) alongside a decrease in ∆*lasR* strain RhlR/I-dependent AHL activity in coculture with the AHL-sensing reporter strain (Fig. S3C and D). Collectively, these data support a model in which pyochelin production by the ∆*lasR* strain is induced and required for pyocyanin-promoting interactions with the WT through initiation of a low-iron response (Fig. 3G).

10.1128/mBio.01865-20.5 FIG S5 Fitness of the ∆*lasR* strain lacking one or both major siderophore biosynthesis pathways. Final proportion of untagged colony forming units was quantified after a 16-h competition with the WT *att*::*lacZ* strain. The dotted line indicates the 0.5 initial proportion (P_i_). Final proportions (P_f_) for the ∆*lasR* wild-type (WT) strains and the ∆*lasR* strain complemented with the *lasR* gene at the native locus (∆*lasR* strain) on LB medium (white background), for the ∆*lasR* strain on LB medium supplemented with 10 μM FeSO_4_ (grey background), and for siderophore-deficient ∆*lasR* mutant derivatives. a versus b, *P* < 0.0006; a versus c, *P* < 0.0001, as determined by one-way analysis of variance for comparison to the ∆*lasR* strain on LB medium with a Sidak multiple-hypotheses correction for *n* ≥ 3 experiments. Download FIG S5, PDF file, 0.1 MB. Copyright © 2020 Mould et al. 2020 Mould et al. This content is distributed under the terms of the Creative Commons Attribution 4.0 International license.

10.1128/mBio.01865-20.6 FIG S6 Pyochelin-containing extracts are biologically active. (A) Absorbance spectra (230 to 600 nm) of extracts from supernatants of the pyochelin-producing ∆*pvdA* strain (solid line) and the pyochelin-deficient ∆*pvdA* ∆*pchE* strain (dotted line) in a 50/50 MeOH/distilled H_2_O solution. Grey vertical lines indicate reported peaks for purified, iron-free pyochelin at 248 and 313 nm. (B) Indicated extracts spotted on chrome azurol S (CAS) agar with EDTA metal chelator as a positive control wherein a change from blue to yellow indicates iron-chelating capacity. CAS activity for the extracts from ∆*pvdA* (+PCH) and ∆*pvdA* ∆*pchE* (NEG) strains are shown. Download FIG S6, PDF file, 0.1 MB. Copyright © 2020 Mould et al. 2020 Mould et al. This content is distributed under the terms of the Creative Commons Attribution 4.0 International license.

### In coculture with the WT, the ∆*lasR* strain responds to citrate, a pyochelin-inducible metabolite.

Many of the upregulated genes in the ∆*lasR* strain upon coculture with the WT have annotations related to organic acids, such as anthranilate and citrate (Table S1 and Fig. S7A). Several lines of evidence suggest that anthranilate was not the factor that stimulated RhlR activity and pyocyanin production in coculture. First, anthranilate supplementation (up to ∼15 mM) did not alter ∆*lasR* strain phenazine production (Fig. S7B). Further, cocultures of the ∆*lasR* mutant with the anthranilate synthase mutant ∆*phnAB* strain, with reduced extracellular anthranilate (Fig. S7C), or with the ∆*pqsA* mutant (Fig. 2C), which accumulates it (54), did not alter coculture phenazine production. Anthranilate is also generated from tryptophan catabolism through the kynurenine pathway (55). Given that coculture pyocyanin production could occur in the absence of exogenous amino acids (Fig. S1A), we concluded that the kynurenine pathway was likely not involved. Together, these data suggested that anthranilate was not a stimulating metabolite.

10.1128/mBio.01865-20.7 FIG S7 Anthranilate (AA) supplementation and loss of potential di- and tricarboxylic acid transporters *dctA* and PA14_51300 did not alter pyocyanin production of the ∆*lasR* strain. (A) Volcano plot indicating anthranilate metabolism gene expression (orange) in the ∆*lasR* strain grown in coculture with the WT. (B) Colony morphology and pyocyanin production are not different upon anthranilate supplementation across a gradient of concentrations. (C) RhlR-dependent pyocyanin production of the ∆*lasR* mutant and the anthranilate synthase mutant (∆*phnAB* strain) in mono- and cocultures on LB medium after 18 h. (D) The major succinate, fumarate, and malate transporter *dctA* was dispensable in the ∆*lasR* strain background for RhlR-dependent pyocyanin production in coculture with the WT. (E) The broad TCA cycle intermediate transporter PA14_51300 was not required in the ∆*lasR* strain for pyocyanin production in coculture with the WT. Download FIG S7, PDF file, 0.5 MB. Copyright © 2020 Mould et al. 2020 Mould et al. This content is distributed under the terms of the Creative Commons Attribution 4.0 International license.

**FIG 4:**
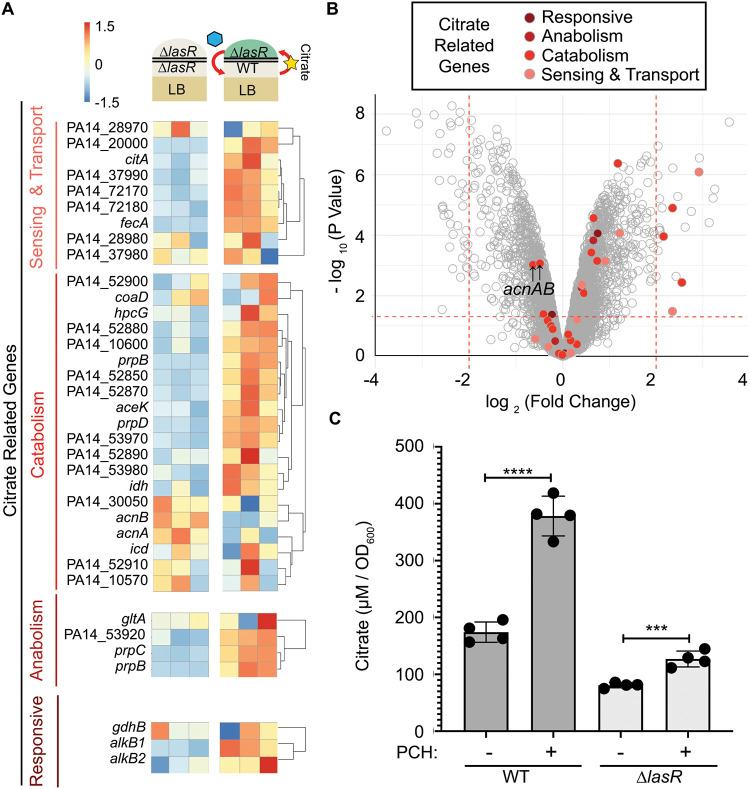
Citrate release by WT is induced by pyochelin exposure. (A) Subset of expression data in ∆*lasR* cocultures (Fig. 3A shows the setup) for genes annotated as being involved in citrate sensing, transport, catabolism, anabolism, and those shown to be responsive to citrate. (B) Volcano plot of expression data of ∆*lasR* cocultures with each point representing the log_2_(∆*lasR* cells grown on WT/∆*lasR* cells grown on ∆*lasR* cells) expression and –log_10_(*P* value) of a single gene. Genes shown in panel A are highlighted in color. (C) Citrate concentrations in supernatants from wild-type and ∆*lasR* stationary-phase cultures after growth in LB medium supplemented with extracts containing 50 μM pyochelin (PCH+) or an equal volume of control extracts (PCH−). A representative experiment with four biological replicates repeated 3 independent days is shown. ***, *P* < 0.0005; ****, *P* ≤ 0.0001, as determined by two-tailed *t* tests of paired ratios.

In light of the observation that 20% of the most strongly differentially expressed genes [|log_2_ (fold change)| of ≥2 with a *P* value of <0.05] were implicated in citrate sensing, transport, catabolism, and anabolism, as annotated by UniProt (56) and www.pseudomonas.com (57), we looked at all genes with annotations related to citrate to identify broad expression patterns (Fig. 4A). Among the genes induced in ∆*lasR*/WT cocultures [|log _2_ (fold change)| of ≥0 with a *P* value of <0.05] were genes annotated as citrate responsive or playing roles in citrate sensing and transport or metabolism, with the most strongly upregulated citrate genes involved in sensing, transport, and catabolism specifically (Fig. 4B).

We measured citrate in the supernatants of WT and ∆*lasR* LB cultures based on the following observations: (i) ∆*lasR* strains induced low-iron-responsive genes when grown near the WT but not itself; (ii) ∆*lasR* strain pyochelin production was necessary for coculture interactions that led to increased pyocyanin; (iii) citrate sensing and catabolism genes were induced in ∆*lasR* cells by the presence of WT cells; and (iv) numerous microbes, including Pseudomonas putida, were shown to secrete citrate and other organic acids when iron limited (44, 58–60). Citrate was detected in both WT and ∆*lasR* strain LB culture supernatants (Fig. 4C), and amendment with extracts containing 50 μM pyochelin increased extracellular citrate concentrations by ∼2-fold in WT cultures compared to levels in cultures supplemented with extracts lacking pyoverdine and pyochelin, with a much smaller stimulation in ∆*lasR* cultures under the same conditions (Fig. 4C). This suggested that WT-produced citrate may be involved in WT/∆*lasR* coculture interactions and that citrate release was enhanced by pyochelin produced by the ∆*lasR* strain.

### Citrate induces RhlR-dependent activity and RhlI levels in a ClpX protease-dependent manner in ∆*lasR* cells.

**FIG 5:**
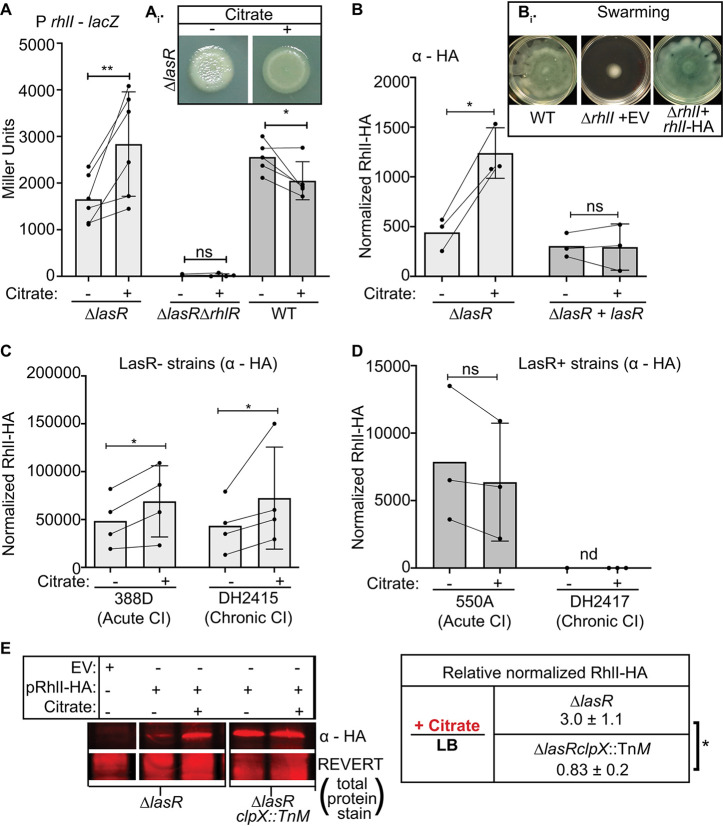
Citrate induced RhlR-dependent *rhlI* promoter activity and stabilized RhlI protein in LasR− strains in a ClpX protease-dependent manner. (A) β-Galactosidase activity for ∆*lasR*, ∆*lasR* ∆*rhlR*, and wild-type strains harboring *att*::P*rhlI-lacZ* on LB medium with or without 20 mM citrate at 24 h. Each point is the average of three biological replicates from 3 to 4 independent experiments. ns, not significant; *, *P* < 0.05; **, *P* < 0.005, as determined by one-way analysis of variance with Dunnett’s multiple hypotheses correction of the indicated comparisons. The inset (A_i_) shows a representative image of ∆*lasR* colony morphology on LB medium with and without 20 mM citrate at 24 h. (B) RhlI-HA protein signal normalized to Revert total protein stain (LiCor) on LB medium with and without 20 mM citrate for the ∆*lasR* strain and *lasR* complemented at the native locus (∆*lasR* strain). ns, not significant; *, *P* < 0.05; **, *P* < 0.005, as determined by analysis of variance with Dunnett’s multiple hypothesis correction for *n* = 3 biological replicates performed on three independent days. The inset (B_i_) illustrates that plasmid-borne RhlI-HA, but not the empty vector (EV), complemented an ∆*rhlI* mutant for swarming. (C) RhlI-HA protein levels on LB medium with and without 20 mM citrate of LasR loss-of-function (LasR−) clinical isolates (CIs) from acute corneal (388D) and chronic CF (DH2415) Infections. *, *P* < 0.05, as determined by two-tailed *t* tests of paired ratios for *n* = 3 experiments for each isolate. (D) RhlI-HA protein levels on LB medium with and without 20 mM citrate of an LasR+ acute corneal CI (550A) of the same multilocus sequence type as 388D and LasR+ chronic CF CI (DH2417) from which DH2415 evolved. ns, not significant as determined by two-tailed *t* tests of paired ratios for *n* = 3 experiments for each isolate. nd, not detected. (E) Representative image and quantification of replicates for the anti-HA antibody analysis of ∆*lasR* and ∆*lasR clpX* Tn::*M* strains carrying a plasmid expressing RhlI-HA or an empty vector (pRhlI-HA or pEV, respectively) grown in LB medium with or without 20 mM citrate. *, *P* < 0.05. as determined by two-tailed *t* tests of paired ratios for *n* = 3 replicates from 3 independent days.

To determine if citrate was sufficient to stimulate RhlR activity in ∆*lasR* cells, we analyzed its effects on *rhlI* promoter fusion activity, colony morphology, and RhlI protein levels. We found that citrate increased *rhlI* promoter activity (P*rhlI*) in ∆*lasR* cells and that its effects were dependent on the presence of RhlR (Fig. 5A). Citrate was sufficient to promote increases in colony pigmentation and colony smoothness, previously characterized to be RhlR-mediated in ∆*lasR* cells (29) (Fig. 5A, inset). In contrast, citrate caused a small but significant reduction in WT P*rhlI* activity compared to that the LB control (Fig. 5A).

To determine if RhlI protein levels were influenced by citrate, we utilized an arabinose-inducible *rhlI-*hemagglutinin (HA) construct to assess RhlI protein levels and stability in the absence and presence of citrate independent of RhlR transcriptional control. RhlI-HA was functional as swarming defects of the ∆*rhlI* mutant were complemented upon expression of RhlI-HA but not by the empty vector (Fig. 5B, inset). RhlI-HA protein levels were 3-fold higher in the ∆*lasR* strain upon citrate supplementation than in the controls (Fig. 5B). Consistent with the absence of an increase in *rhlI* promoter activity in WT strains (Fig. 5A), RhlI-HA protein levels were not higher with citrate in the ∆*lasR* complemented strain (∆*lasR* + *lasR* strain) (Fig. 5B). The differential responses to citrate were also observed in LasR− and LasR+ pairs of clinical isolates (CIs). LasR− CIs from acute (strain 388D) and chronic (strain DH2415) infections had RhlI-HA levels 1.4- and 1.7-fold higher, respectively, in the presence of citrate (Fig. 5C), whereas alterations in RhlI-HA protein levels in LasR+ CIs from acute (strain 550A) or chronic (strain DH2417) infections were not observed (Fig. 5D). Through this work, we successfully identified citrate as a molecule in coculture that specifically promoted RhlI protein levels in LasR− strains, but not in LasR+ strains, by posttranscriptional control. In an attempt to identify transporters involved in the ∆*lasR* strain response to citrate and/or other coculture metabolites, we deleted two organic acid transporters: *dctA* (61) and PA14_51300 (62) in the ∆*lasR* strain background. We found that the ∆*lasR* ∆*dctA* strain showed induction of pyocyanin when it was cocultured with the WT and that induction was dependent on RhlR (Fig. S7D). Similar results were obtained with the ∆*lasR* ∆PA14_51300 strain (Fig. S7E), suggesting that these transporters were not required for the interaction, perhaps due to redundant functions of other proteins or the involvement of other import mechanisms.

The temporal pattern of activation and the stimulation of RhlI-HA in the absence of RhlR control suggested that the RhlI protein may precede signal amplification via the QS transcriptional network. This would be consistent with a primary effect on posttranscriptional modulation of RhlI-mediated RhlR activity, as has been reported previously to occur through an RhlS small RNA (sRNA)-dependent mechanism (63); however, we found no apparent difference in RhlS expression levels in our RNA-seq reads in coculture. To begin to unravel the mechanisms by which citrate promoted RhlR/I-dependent signaling and RhlI stability in ∆*lasR* cells, we analyzed the role of two proteases previously found to target and degrade RhlI (i.e., Lon and ClpXP) (64). Given that knockouts of Lon protease have a less substantial rise in RhlR/I expression in ∆*lasR* strains than in the WT (65), we focused on the role of ClpXP in ∆*lasR* cells. We found that citrate induction of RhlI-HA protein levels in the ∆*lasR* strain relative to that in the LB control was dependent on functional ClpX protease (Fig. 5E). More specifically, when ClpX, a protease shown to degrade RhlI, is nonfunctional (i.e., ∆*lasR clpX*::Tn*M* strain), RhlI-HA levels did not increase on LB plus citrate relative to that of the LB control, unlike the level in the ∆*lasR* strain comparator (Fig. 5E). Under LB culture conditions, RhlI-HA levels were 3.20- ± 2.1-fold higher in the ∆*lasR clpX*::Tn*M* strain than in the ∆*lasR* strain, which mirrors the 3-fold induction observed for the ∆*lasR* strain on LB medium with or without citrate. Under citrate-supplemented conditions, no significant difference in RhlI-HA levels was observed for the ∆*lasR clpX*::Tn*M* relative to that in the ∆*lasR* strain (fold change of 1.01 ± 0.53). In other words, as previously noted for WT strains, ClpX may degrade RhlI in ∆*lasR* cells and play a role in the ∆*lasR* cell response to citrate. The distinct responses and mechanisms identified between LasR+ and LasR− strains under iron limitation and exposure to the low-iron-associated molecules, citrate and pyochelin, enabled increases in antagonistic factors beyond monoculture levels as an emergent property of P. aeruginosa intraspecies interactions.

## DISCUSSION

**FIG 6:**
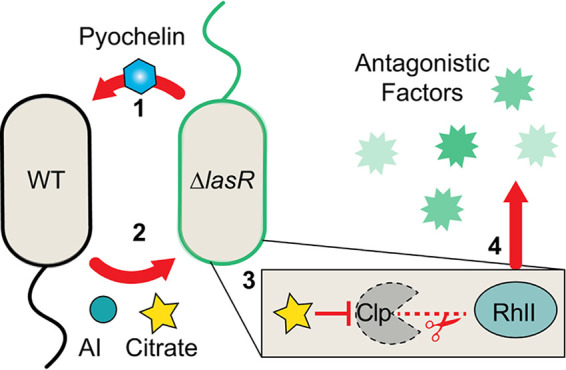
Model for wild-type and ∆*lasR* coculture interactions. ∆*lasR* strain-produced pyochelin promotes citrate release in the wild type (1). Citrate (and diffusible autoinducer) released by the wild type in coculture stimulates RhlR/I-dependent activity in a ∆*lasR* strain (2). Citrate stabilizes RhlI protein in ∆*lasR* cells potentially through a ClpXP protease-dependent mechanism (3), which ultimately promotes the production of antagonistic factors like pyocyanin toxin and rhamnolipid surfactant above monoculture levels (4).

In this study, we described an emergent outcome of coculturing LasR− and LasR+ strains of P. aeruginosa in which their interactions promoted the toxic exoproducts pyocyanin and rhamnolipids (Fig. 6 provides a model). We determined that, in coculture, the ∆*lasR* strain produces the siderophore pyochelin and that exogenous pyochelin induced citrate release more strongly in the WT than in ∆*lasR* strain. Citrate increased RhlI protein levels and induced RhlR-dependent activity only in ∆*lasR* cells and not WT cells (Fig. 6). Western blot analyses of RhlI-HA expressed from a regulated promoter led us to propose that the increase in RhlR-dependent signaling is due to decreased degradation of RhlI by ClpXP, a known negative regulator of RhlI (65, 66), or through other mechanisms of posttranscriptional regulation. The differences in siderophore production, citrate release, and RhlR/I-dependent activation between P. aeruginosa LasR+ and LasR− strains in coculture reflect the pronounced differences between strains that drive QS reactivation. Previous studies have shown that LasR− strain colony morphology and phenazine production change in the presence of other species such as Candida albicans (29) and Staphylococcus aureus (see Fig. 3B in reference 67), and future work will determine if pyochelin and citrate also participate in these interspecies interactions as many microbial interactions have been shown to be influenced by iron availability (68–71). Furthermore, the induction of RhlR activity that can occur in late-stationary-phase ∆*lasR* cultures (30, 72) may relate to changes in iron or TCA cycle intermediates. While we found that WT-produced autoinducers, including 3OC12HSL, C4HSL, and PQS, were not required for coculture stimulation, they clearly contributed to the enhanced RhlR-dependent activity, which is consistent with intercolony QS interactions demonstrated previously (73).

The stimulatory relationship between LasR+ and LasR− strains was remarkably stable as it was observed when strains were mixed within single spot colonies (Fig. 1A) and when strains were separated by either filters (Fig. 3) or millimeter distances on an agar plate (Fig. 2B). The LasR−/LasR+ interactions occurred across distinct media (see Fig. S1A in the supplemental material), among genetically diverse LasR+ and LasR− clinical isolates (Fig. S1B), and over a wide range of relative proportions of each type (Fig. 1C). Of note, colonies inoculated at a 80:20 WT-to-∆*lasR* cell ratio had more zones with the *lasR* mutant-associated phenotypes described as sheen and lysis than colonies with a 20:80 WT-to-∆*lasR* cell ratio (Fig. 1C). At both ratios, ∆*lasR* cell numbers increased slightly relative to level of the wild type (Fig. 1D). We propose that the reduced appearance of sheen and lysis in mixed colonies inoculated with more ∆*lasR* cells reflects a requirement for a sufficient proportion of ∆*lasR* cells to initiate the WT-∆*lasR* cell interactions that activate RhlR and restore a more WT-like phenotype to LasR− cells. Furthermore, if RhlR signaling is not fully activated in ∆*lasR* cells, there may be regions of increased ∆*lasR* cell killing via WT-produced factors such as cyanide (74).

The consequences of this intraspecies interaction may explain the worse outcomes exhibited by patients in which LasR− strains are detected (13), but future studies that include genotypes, monoculture and coculture phenotypes, and longitudinal outcome data will be required. RhlR plays other important roles in host interactions (75) which may benefit P. aeruginosa LasR− strains. The observation that *rhlR* mutants are rare in natural isolates and that LasR− strains with active RhlR are virulent (25, 76) underscores the relevance of this mechanism and highlights the importance of understanding how microbial interactions influence RhlR activity.

As studies of inter- and intraspecies interactions progress, it is becoming increasingly clear that the environment can dictate the outcome of microbial interactions (77). In fact, even the importance of QS regulation for fitness depends on nutrient sources and conditions (78, 79). As ∆*lasR* cell-produced pyochelin was a key component of the interaction and as pyochelin production is repressed under conditions of excess iron, it was not surprising that iron supplementation suppressed the interaction (Fig. 3F and Fig. S3C and D) without significantly altering the final colony CFU count or strain ratios relative to those of the LB control (Fig. S4). Siderophore-mediated iron uptake is often required *in vivo* (34, 80, 81) due to iron sequestration by host proteins (82–85); thus, *in vivo* settings may support these interactions.

Interestingly, pyoverdine, the higher-affinity siderophore, was not required for the coculture response, mirroring findings that genes for biosynthesis of pyoverdine, but not pyochelin, are commonly disrupted in isolates from chronic CF patients (39–41). In the absence of pyoverdine (i.e., the ∆*lasR ∆pvdA* strain), we observed more pyocyanin in coculture with the WT than with the ∆*lasR* strain (Fig. 3D), and we speculate that this is due to increased pyochelin production by ∆*pvdA* cells, but future studies will be required to test this model. It was interesting to find that in WT/∆*lasR* coculture, heme-related proteins, *hasAP*, *hasS*, and *hasD*, were among the top eight most-upregulated genes by the ∆*lasR* strain because the presence of *lasR* mutants and heme content are both reported biomarkers of disease progression in CF patients (13, 86). Coculture-induced *lasR* mutant phenotypes may link these two correlative observations.

Citrate, a TCA intermediate released under iron limitation as a result of overflow metabolism (43, 44, 60, 87), can be used by P. aeruginosa and other microbes for iron acquisition due to its iron chelating properties (88). The increased siderophore production by ∆*lasR* cells in coculture likely reflects different metabolic strategies between genotypes. Ongoing work will investigate the mechanisms that drive differences in metabolism and iron requirements in order to determine how these differences shape microbial and host interactions. It is likely that Crc-mediated catabolite repression is involved in the response to citrate and the control of RhlI levels (64, 66). That a mechanism exists for the induction of RhlR-mediated QS in response to citrate secreted when iron is limiting dovetails with reports of increased expression of the P. aeruginosa QS regulon in low iron in LasR+ cells (89–91). This coordinate regulation may aid in iron acquisition as QS-controlled phenazines, such as pyocyanin, reduce poorly soluble Fe^3+^ to Fe^2+^ and facilitate its uptake via the Feo system (92). Additionally, rhamnolipids have been employed for iron remediation (93, 94), which suggests that their surfactant activity may increase P. aeruginosa substrate iron uptake in part through hydroxy-alkyl-quinolone-dependent mechanisms (95).

Given that anthranilate did not alter ∆*lasR* colony morphology or phenazine production, we did not further investigate anthranilate as a cross-fed metabolite involved in WT-∆*lasR* cell interactions. We speculate that the increased expression of anthranilate catabolism genes in coculture may be more reflective of increases in RhlR activity than increased exposure to anthranilate given reports highlighting RhlR activation of *antABC* and *catABC* anthranilate catabolism genes (59).

As the presence of heterogeneous genotypes within single-species populations becomes increasingly appreciated, it is important to understand how commonly encountered genotypes interact to influence population-level behavior. Other work shows that cocultures can influence the survival of other genotypes (96, 97). Here, we show that intergenotype interactions lead to increased RhlR-dependent signaling in LasR− strains. It is likely that a wide array of such interactions has yet to be uncovered.

## MATERIALS AND METHODS

### Strains and growth conditions.

Strains used in this study are listed in Table S2 in the supplemental material. Bacteria were maintained on LB (lysogeny broth) medium with 1.5% agar. Saccharomyces cerevisiae strains for cloning were maintained on yeast-peptone-dextrose (YPD) medium with 2% agar. With the exception of pyochelin complementation experiments, which were performed in 12-well dishes with a 2-ml total volume containing 50 μM pyochelin (or an equal-volume negative-control extract), colony biofilm assays were performed in 100-mm petri dishes with a 25-ml total volume. Where stated, a 20 mM concentration of the indicated metabolite was added to the medium (liquid or molten agar). Planktonic cultures were grown on roller drums at 37°C. Artificial sputum medium (ASM) was made as described previously (27).

10.1128/mBio.01865-20.9 TABLE S2 Strains and plasmids used in this study. Download Table S2, DOCX file, 0.1 MB. Copyright © 2020 Mould et al. 2020 Mould et al. This content is distributed under the terms of the Creative Commons Attribution 4.0 International license.

### Competition assays.

Competition assays were performed to determine the relative fitness of P. aeruginosa mutants. Strains to be competed were grown overnight and adjusted to an optical density at 600 nm (OD_600_) of 1. Competing strains were combined with the PA14 *att*::*lacZ* strain in a 1:1 ratio unless otherwise stated. Following a 15-s vortex, 5 μl of the combined suspension was spotted on LB agar. After 16 h, colony biofilms (and agar) were cored, placed in 1.5-ml tubes with 500 μl of LB, and agitated vigorously for 5 min using a Genie Disruptor (Zymo). This suspension was diluted, spread on LB plates supplemented with 150 μg/ml 5-bromo-4-chloro-3-indolyl-d-galactopyranoside (X-Gal) using glass beads, and incubated at 37°C until blue colonies were visible (∼24 h). The numbers of blue and white colonies per plate were counted, and the final proportions were recorded. Each competition was run in triplicate on 3 separate days.

### Additional methods.

See Text S1 in the supplemental material for methods describing plasmid construction, pyocyanin quantification, colony proximity image analysis, swarming, RNA collection and processing, pyochelin extraction and quantification, citrate quantification, β-galactosidase quantification, Western blotting, and acyl-homoserine lactone activity assays.

10.1128/mBio.01865-20.10 TEXT S1 Methods describing plasmid construction, pyocyanin quantification, colony proximity image analysis, swarming, RNA collection and processing, pyochelin extraction and quantification, citrate quantification, β-galactosidase quantification, Western blotting, and acyl-homoserine lactone activity assays. Download Text S1, DOCX file, 0.03 MB. Copyright © 2020 Mould et al. 2020 Mould et al. This content is distributed under the terms of the Creative Commons Attribution 4.0 International license.

### Data availability.

Data for RNA-seq analysis of P. aeruginosa ∆*lasR* grown on the ∆*lasR* or WT strain in coculture has been uploaded to the Gene Expression Omnibus (GEO) repository (https://www.ncbi.nlm.nih.gov/geo/) under accession number GSE149385.

## References

[1] Demers EG, Biermann AR, Masonjones S, Crocker AW, Ashare A, Stajich JE, Hogan DA 2018 Evolution of drug resistance in an antifungal-naive chronic *Candida lusitaniae* infection. Proc Natl Acad Sci U S A 115:12040–12045. doi:10.1073/pnas.1807698115. 30389707 PMC6255150

[2] Boles BR, Thoendel M, Singh PK 2004 Self-generated diversity produces "insurance effects" in biofilm communities. Proc Natl Acad Sci U S A 101:16630–16635. doi:10.1073/pnas.0407460101. 15546998 PMC528905

[3] Zhao S, Lieberman TD, Poyet M, Kauffman KM, Gibbons SM, Groussin M, Xavier RJ, Alm EJ 2019 Adaptive evolution within gut microbiomes of healthy people. Cell Host Microbe 25:656–667.e8. doi:10.1016/j.chom.2019.03.007. 31028005 PMC6749991

[4] Azimi S, Roberts AEL, Peng S, Weitz JS, McNally A, Brown SP, Diggle SP 2020 Allelic polymorphism shapes community function in evolving *Pseudomonas aeruginosa* populations. ISME J 14:1929–1942. doi:10.1038/s41396-020-0652-0. 32341475 PMC7368067

[5] Bos LD, Meinardi S, Blake D, Whiteson K 2016 Bacteria in the airways of patients with cystic fibrosis are genetically capable of producing VOCs in breath. J Breath Res 10:047103. doi:10.1088/1752-7163/10/4/047103. 27991430

[6] Jorgensen KM, Wassermann T, Johansen HK, Christiansen LE, Molin S, Hoiby N, Ciofu O 2015 Diversity of metabolic profiles of cystic fibrosis *Pseudomonas aeruginosa* during the early stages of lung infection. Microbiology 161:1447–1462. doi:10.1099/mic.0.000093. 25873584

[7] Markussen T, Marvig RL, Gómez-Lozano M, Aanæs K, Burleigh AE, Høiby N, Johansen HK, Molin S, Jelsbak L 2014 Environmental heterogeneity drives within-host diversification and evolution of *Pseudomonas aeruginosa*. mBio 5:e01592-14. doi:10.1128/mBio.01592-14. 25227464 PMC4172072

[8] Wilder CN, Allada G, Schuster M 2009 Instantaneous within-patient diversity of *Pseudomonas aeruginosa* quorum sensing populations from cystic fibrosis lung infections. Infect Immun 77:5631–5639. doi:10.1128/IAI.00755-09. 19805523 PMC2786440

[9] Winstanley C, O'Brien S, Brockhurst MA 2016 *Pseudomonas aeruginosa* evolutionary adaptation and diversification in cystic fibrosis chronic lung infections. Trends Microbiol 24:327–337. doi:10.1016/j.tim.2016.01.008. 26946977 PMC4854172

[10] Woo TE, Duong J, Jervis NM, Rabin HR, Parkins MD, Storey DG 2016 Virulence adaptations of *Pseudomonas aeruginosa* isolated from patients with non-cystic fibrosis bronchiectasis. Microbiology 162:2126–2135. doi:10.1099/mic.0.000393. 27902425 PMC5410107

[11] Workentine ML, Sibley CD, Glezerson B, Purighalla S, Norgaard-Gron JC, Parkins MD, Rabin HR, Surette MG 2013 Phenotypic heterogeneity of *Pseudomonas aeruginosa* populations in a cystic fibrosis patient. PLoS One 8:e60225. doi:10.1371/journal.pone.0060225. 23573242 PMC3616088

[12] Hammond JH, Hebert WP, Naimie A, Ray K, Van Gelder RD, DiGiandomenico A, Lalitha P, Srinivasan M, Acharya NR, Lietman T, Hogan DA, Zegans ME 2016 Environmentally endemic *Pseudomonas aeruginosa* strains with mutations in *lasR* are associated with increased disease severity in corneal ulcers. mSphere 1:e00140-16. doi:10.1128/mSphere.00140-16. 27631025 PMC5014915

[13] Hoffman LR, Kulasekara HD, Emerson J, Houston LS, Burns JL, Ramsey BW, Miller SI 2009 *Pseudomonas aeruginosa lasR* mutants are associated with cystic fibrosis lung disease progression. J Cyst Fibros 8:66–70. doi:10.1016/j.jcf.2008.09.006. 18974024 PMC2631641

[14] Smith EE, Buckley DG, Wu Z, Saenphimmachak C, Hoffman LR, D'Argenio DA, Miller SI, Ramsey BW, Speert DP, Moskowitz SM, Burns JL, Kaul R, Olson MV 2006 Genetic adaptation by *Pseudomonas aeruginosa* to the airways of cystic fibrosis patients. Proc Natl Acad Sci U S A 103:8487–8492. doi:10.1073/pnas.0602138103. 16687478 PMC1482519

[15] Cabrol S, Olliver A, Pier GB, Andremont A, Ruimy R 2003 Transcription of quorum-sensing system genes in clinical and environmental isolates of *Pseudomonas aeruginosa*. J Bacteriol 185:7222–7230. doi:10.1128/JB.185.24.7222-7230.2003. 14645283 PMC296264

[16] Denervaud V, TuQuoc P, Blanc D, Favre-Bonte S, Krishnapillai V, Reimmann C, Haas D, van Delden C 2004 Characterization of cell-to-cell signaling-deficient *Pseudomonas aeruginosa* strains colonizing intubated patients. J Clin Microbiol 42:554–562. doi:10.1128/jcm.42.2.554-562.2004. 14766816 PMC344450

[17] Lee J, Zhang L 2015 The hierarchy quorum sensing network in *Pseudomonas aeruginosa*. Protein Cell 6:26–41. doi:10.1007/s13238-014-0100-x. 25249263 PMC4286720

[18] Kanthakumar K, Taylor G, Tsang KW, Cundell DR, Rutman A, Smith S, Jeffery PK, Cole PJ, Wilson R 1993 Mechanisms of action of *Pseudomonas aeruginosa* pyocyanin on human ciliary beat *in vitro*. Infect Immun 61:2848–2853. doi:10.1128/IAI.61.7.2848-2853.1993. 8390405 PMC280930

[19] Wilson R, Pitt T, Taylor G, Watson D, MacDermot J, Sykes D, Roberts D, Cole P 1987 Pyocyanin and 1-hydroxyphenazine produced by *Pseudomonas aeruginosa* inhibit the beating of human respiratory cilia *in vitro*. J Clin Invest 79:221–229. doi:10.1172/JCI112787. 3098783 PMC424027

[20] Wilson R, Roberts D, Cole P 1985 Effect of bacterial products on human ciliary function *in vitro*. Thorax 40:125–131. doi:10.1136/thx.40.2.125. 3919460 PMC460002

[21] Blumer C, Haas D 2000 Iron regulation of the *hcnABC* genes encoding hydrogen cyanide synthase depends on the anaerobic regulator ANR rather than on the global activator GacA in *Pseudomonas fluorescens* CHA0. Microbiology 146:2417–2424. doi:10.1099/00221287-146-10-2417. 11021918

[22] Howe J, Bauer J, Andrä J, Schromm AB, Ernst M, Rössle M, Zähringer U, Rademann J, Brandenburg K 2006 Biophysical characterization of synthetic rhamnolipids. FEBS J 273:5101–5112. doi:10.1111/j.1742-4658.2006.05507.x. 17059466

[23] Ortiz A, Teruel JA, Espuny MJ, Marqués A, Manresa Á, Aranda FJ 2006 Effects of dirhamnolipid on the structural properties of phosphatidylcholine membranes. Int J Pharm 325:99–107. doi:10.1016/j.ijpharm.2006.06.028. 16872765

[24] Moussa Z, Chebl M, Patra D 2017 Interaction of curcumin with 1,2-dioctadecanoyl-sn-glycero-3-phosphocholine liposomes: intercalation of rhamnolipids enhances membrane fluidity, permeability and stability of drug molecule. Colloids Surf B Biointerfaces 149:30–37. doi:10.1016/j.colsurfb.2016.10.002. 27716529

[25] Feltner JB, Wolter DJ, Pope CE, Groleau MC, Smalley NE, Greenberg EP, Mayer-Hamblett N, Burns J, Deziel E, Hoffman LR, Dandekar AA 2016 LasR variant cystic fibrosis isolates reveal an adaptable quorum-sensing hierarchy in *Pseudomonas aeruginosa*. mBio 7:e01513-16. doi:10.1128/mBio.01513-16. 27703072 PMC5050340

[26] D'Argenio D, Wu M, Hoffman L, Kulasekara H, Déziel E, Smith E, Nguyen H, Ernst R, Larson Freeman T, Spencer D, Brittnacher M, Hayden H, Selgrade S, Klausen M, Goodlett D, Burns J, Ramsey B, Miller S 2007 Growth phenotypes of *Pseudomonas aeruginosa lasR* mutants adapted to the airways of cystic fibrosis patients. Mol Microbiol 64:512–533. doi:10.1111/j.1365-2958.2007.05678.x. 17493132 PMC2742308

[27] Clay ME, Hammond JH, Zhong F, Chen X, Kowalski CH, Lee AJ, Porter MS, Hampton TH, Greene CS, Pletneva EV, Hogan DA 2020 *Pseudomonas aeruginosa lasR* mutant fitness in microoxia is supported by an Anr-regulated oxygen-binding hemerythrin. Proc Natl Acad Sci U S A 117:3167–3173. doi:10.1073/pnas.1917576117. 31980538 PMC7022198

[28] Basta DW, Bergkessel M, Newman DK 2017 Identification of fitness determinants during energy-limited growth arrest in *Pseudomonas aeruginosa*. mBio 8:e01170-17. doi:10.1128/mBio.01170-17. 29184024 PMC5705914

[29] Cugini C, Morales DK, Hogan DA 2010 *Candida albicans*-produced farnesol stimulates *Pseudomonas* quinolone signal production in LasR-defective *Pseudomonas aeruginosa* strains. Microbiology 156:3096–3107. doi:10.1099/mic.0.037911-0. 20656785 PMC3068698

[30] Cabeen MT 2014 Stationary phase-specific virulence factor overproduction by a lasR mutant of *Pseudomonas aeruginosa*. PLoS One 9:e88743. doi:10.1371/journal.pone.0088743. 24533146 PMC3923063

[31] Van Delden C, Pesci EC, Pearson JP, Iglewski BH 1998 Starvation selection restores elastase and rhamnolipid production in a *Pseudomonas aeruginosa* quorum-sensing mutant. Infect Immun 66:4499–4502. doi:10.1128/.66.9.4499-4502.1998. 9712807 PMC108545

[32] Ankenbauer RG, Toyokuni T, Staley A, Rinehart KL, Cox CD 1988 Synthesis and biological activity of pyochelin, a siderophore of *Pseudomonas aeruginosa*. J Bacteriol 170:5344–5351. doi:10.1128/jb.170.11.5344-5351.1988. 3141386 PMC211611

[33] Brandel J, Humbert N, Elhabiri M, Schalk IJ, Mislin GL, Albrecht-Gary AM 2012 Pyochelin, a siderophore of *Pseudomonas aeruginosa*: physicochemical characterization of the iron(III), copper(II) and zinc(II) complexes. Dalton Trans 41:2820–2834. doi:10.1039/c1dt11804h. 22261733

[34] Gi M, Lee KM, Kim SC, Yoon JH, Yoon SS, Choi JY 2015 A novel siderophore system is essential for the growth of *Pseudomonas aeruginosa* in airway mucus. Sci Rep 5:14644. doi:10.1038/srep14644. 26446565 PMC4597187

[35] Cox CD, Adams P 1985 Siderophore activity of pyoverdin for *Pseudomonas aeruginosa*. Infect Immun 48:130–138. doi:10.1128/IAI.48.1.130-138.1985. 3156815 PMC261925

[36] Bhakta MN, Wilks A 2006 The mechanism of heme transfer from the cytoplasmic heme binding protein PhuS to the δ-regioselective heme oxygenase of *Pseudomonas aeruginosa*. Biochemistry 45:11642–11649. doi:10.1021/bi060980l. 16981723 PMC2631378

[37] Wegele R, Tasler R, Zeng Y, Rivera M, Frankenberg-Dinkel N 2004 The heme oxygenase(s)-phytochrome system of *Pseudomonas aeruginosa*. J Biol Chem 279:45791–45802. doi:10.1074/jbc.M408303200. 15310749

[38] Zhou H, Lu F, Latham C, Zander DS, Visner GA 2004 Heme oxygenase-1 expression in human lungs with cystic fibrosis and cytoprotective effects against *Pseudomonas aeruginosa in vitro*. Am J Respir Crit Care Med 170:633–640. doi:10.1164/rccm.200311-1607OC. 15184199

[39] Nguyen AT, O'Neill MJ, Watts AM, Robson CL, Lamont IL, Wilks A, Oglesby-Sherrouse AG 2014 Adaptation of iron homeostasis pathways by a *Pseudomonas aeruginosa* pyoverdine mutant in the cystic fibrosis lung. J Bacteriol 196:2265–2276. doi:10.1128/JB.01491-14. 24727222 PMC4054187

[40] Marvig RL, Damkiær S, Khademi SMH, Markussen TM, Molin S, Jelsbak L 2014 Within-host evolution of *Pseudomonas aeruginosa* reveals adaptation toward iron acquisition from hemoglobin. mBio 5:e00966-14. doi:10.1128/mBio.00966-14. 24803516 PMC4010824

[41] Andersen SB, Marvig RL, Molin S, Krogh Johansen H, Griffin AS 2015 Long-term social dynamics drive loss of function in pathogenic bacteria. Proc Natl Acad Sci U S A 112:10756–10761. doi:10.1073/pnas.1508324112. 26240352 PMC4553784

[42] Oexle H, Gnaiger E, Weiss G 1999 Iron-dependent changes in cellular energy metabolism: influence on citric acid cycle and oxidative phosphorylation. Biochim Biophys Acta 1413:99–107. doi:10.1016/s0005-2728(99)00088-2. 10556622

[43] Carlson RP, Beck AE, Phalak P, Fields MW, Gedeon T, Hanley L, Harcombe WR, Henson MA, Heys JJ 2018 Competitive resource allocation to metabolic pathways contributes to overflow metabolisms and emergent properties in cross-feeding microbial consortia. Biochem Soc Trans 46:269–284. doi:10.1042/BST20170242. 29472366

[44] Sasnow SS, Wei H, Aristilde L 2016 Bypasses in intracellular glucose metabolism in iron-limited *Pseudomonas putida*. Microbiologyopen 5:3–20. doi:10.1002/mbo3.287. 26377487 PMC4767421

[45] Hammond JH, Dolben EF, Smith TJ, Bhuju S, Hogan DA 2015 Links between Anr and quorum sensing in *Pseudomonas aeruginosa* biofilms. J Bacteriol 197:2810–2820. doi:10.1128/JB.00182-15. 26078448 PMC4524035

[46] O'Loughlin CT, Miller LC, Siryaporn A, Drescher K, Semmelhack MF, Bassler BL 2013 A quorum-sensing inhibitor blocks *Pseudomonas aeruginosa* virulence and biofilm formation. Proc Natl Acad Sci U S A 110:17981–17986. doi:10.1073/pnas.1316981110. 24143808 PMC3816427

[47] Lau GW, Hassett DJ, Ran H, Kong F 2004 The role of pyocyanin in *Pseudomonas aeruginosa* infection. Trends Mol Med 10:599–606. doi:10.1016/j.molmed.2004.10.002. 15567330

[48] Whiteley M, Lee KM, Greenberg EP 1999 Identification of genes controlled by quorum sensing in *Pseudomonas aeruginosa*. Proc Natl Acad Sci U S A 96:13904–13909. doi:10.1073/pnas.96.24.13904. 10570171 PMC24163

[49] Brint JM, Ohman DE 1995 Synthesis of multiple exoproducts in *Pseudomonas aeruginosa* is under the control of RhlR-RhlI, another set of regulators in strain PAO1 with homology to the autoinducer-responsive LuxR-LuxI family. J Bacteriol 177:7155–7163. doi:10.1128/jb.177.24.7155-7163.1995. 8522523 PMC177595

[50] Rahme LG, Tan MW, Le L, Wong SM, Tompkins RG, Calderwood SB, Ausubel FM 1997 Use of model plant hosts to identify *Pseudomonas aeruginosa* virulence factors. Proc Natl Acad Sci U S A 94:13245–13250. doi:10.1073/pnas.94.24.13245. 9371831 PMC24294

[51] Köhler T, Curty LK, Barja F, van Delden C, Pechère JC 2000 Swarming of *Pseudomonas aeruginosa* is dependent on cell-to-cell signaling and requires flagella and pili. J Bacteriol 182:5990–5996. doi:10.1128/jb.182.21.5990-5996.2000. 11029417 PMC94731

[52] Mi H, Muruganujan A, Ebert D, Huang X, Thomas PD 2019 PANTHER version 14: more genomes, a new PANTHER GO-slim and improvements in enrichment analysis tools. Nucleic Acids Res 47:D419–D426. doi:10.1093/nar/gky1038. 30407594 PMC6323939

[53] Louden BC, Haarmann D, Lynne AM 2011 Use of blue agar CAS assay for siderophore detection. J Microbiol Biol Educ 12:51–53. doi:10.1128/jmbe.v12i1.249. 23653742 PMC3577196

[54] Calfee MW, Coleman JP, Pesci EC 2001 Interference with *Pseudomonas* quinolone signal synthesis inhibits virulence factor expression by *Pseudomonas aeruginosa*. Proc Natl Acad Sci U S A 98:11633–11637. doi:10.1073/pnas.201328498. 11573001 PMC58781

[55] Farrow JM 3rd, Pesci EC 2007 Two distinct pathways supply anthranilate as a precursor of the *Pseudomonas* quinolone signal. J Bacteriol 189:3425–3433. doi:10.1128/JB.00209-07. 17337571 PMC1855905

[56] The UniProt Consortium. 2018 UniProt: a worldwide hub of protein knowledge. Nucleic Acids Res 47:D506–D515. doi:10.1093/nar/gky1049. PMC6323992 30395287

[57] Winsor GL, Griffiths EJ, Lo R, Dhillon BK, Shay JA, Brinkman FS 2016 Enhanced annotations and features for comparing thousands of *Pseudomonas* genomes in the *Pseudomonas* genome database. Nucleic Acids Res 44:D646–D653. doi:10.1093/nar/gkv1227. 26578582 PMC4702867

[58] Lesuisse E, Simon M, Klein R, Labbe P 1992 Excretion of anthranilate and 3-hydroxyanthranilate by *Saccharomyces cerevisiae*: relationship to iron metabolism. J Gen Microbiol 138:85–89. doi:10.1099/00221287-138-1-85. 1556559

[59] Choi Y, Park HY, Park SJ, Park SJ, Kim SK, Ha C, Im SJ, Lee JH 2011 Growth phase-differential quorum sensing regulation of anthranilate metabolism in *Pseudomonas aeruginosa*. Mol Cells 32:57–65. doi:10.1007/s10059-011-2322-6. 21614486 PMC3887655

[60] Odoni DI, van Gaal MP, Schonewille T, Tamayo-Ramos JA, Martins dos Santos VAP, Suarez-Diez M, Schaap PJ 2017 *Aspergillus niger* secretes citrate to increase iron Bioavailability. Front Microbiol 8:1424. doi:10.3389/fmicb.2017.01424. 28824560 PMC5539119

[61] Valentini M, Storelli N, Lapouge K 2011 Identification of C(4)-dicarboxylate transport systems in *Pseudomonas aeruginosa* PAO1. J Bacteriol 193:4307–4316. doi:10.1128/JB.05074-11. 21725012 PMC3165536

[62] Filiatrault MJ, Tombline G, Wagner VE, Van Alst N, Rumbaugh K, Sokol P, Schwingel J, Iglewski BH 2013 *Pseudomonas aeruginosa* PA1006, which plays a role in molybdenum homeostasis, is required for nitrate utilization, biofilm formation, and virulence. PLoS One 8:e55594. doi:10.1371/journal.pone.0055594. 23409004 PMC3568122

[63] Thomason MK, Voichek M, Dar D, Addis V, Fitzgerald D, Gottesman S, Sorek R, Greenberg EP 2019 A *rhlI* 5′ UTR-derived sRNA regulates RhlR-dependent quorum sensing in *Pseudomonas aeruginosa*. mBio 10:e02253-19. doi:10.1128/mBio.02253-19. 31594819 PMC6786874

[64] Yang N, Ding S, Chen F, Zhang X, Xia Y, Di H, Cao Q, Deng X, Wu M, Wong CC, Tian XX, Yang CG, Zhao J, Lan L 2015 The Crc protein participates in down-regulation of the Lon gene to promote rhamnolipid production and Rhl quorum sensing in *Pseudomonas aeruginosa*. Mol Microbiol 96:526–547. doi:10.1111/mmi.12954. 25641250

[65] Takaya A, Tabuchi F, Tsuchiya H, Isogai E, Yamamoto T 2008 Negative regulation of quorum-sensing systems in *Pseudomonas aeruginosa* by ATP-dependent Lon protease. J Bacteriol 190:4181–4188. doi:10.1128/JB.01873-07. 18408026 PMC2446771

[66] Yang N, Lan L 2016 *Pseudomonas aeruginosa* Lon and ClpXP proteases: roles in linking carbon catabolite repression system with quorum-sensing system. Curr Genet 62:1–6. doi:10.1007/s00294-015-0499-5. 26045103

[67] Hoffman LR, Richardson AR, Houston LS, Kulasekara HD, Martens-Habbena W, Klausen M, Burns JL, Stahl DA, Hassett DJ, Fang FC, Miller SI 2010 Nutrient availability as a mechanism for selection of antibiotic tolerant *Pseudomonas aeruginosa* within the CF airway. PLoS Pathog 6:e1000712. doi:10.1371/journal.ppat.1000712. 20072604 PMC2795201

[68] Harrison F, Paul J, Massey RC, Buckling A 2008 Interspecific competition and siderophore-mediated cooperation in *Pseudomonas aeruginosa*. ISME J 2:49–55. doi:10.1038/ismej.2007.96. 18180746

[69] Lopez-Medina E, Fan D, Coughlin LA, Ho EX, Lamont IL, Reimmann C, Hooper LV, Koh AY 2015 *Candida albicans* inhibits *Pseudomonas aeruginosa* virulence through suppression of pyochelin and pyoverdine biosynthesis. PLoS Pathog 11:e1005129. doi:10.1371/journal.ppat.1005129. 26313907 PMC4552174

[70] Weaver VB, Kolter R 2004 *Burkholderia spp*. alter *Pseudomonas aeruginosa* physiology through iron sequestration. J Bacteriol 186:2376–2384. doi:10.1128/jb.186.8.2376-2384.2004. 15060040 PMC412164

[71] Scott JE, Li K, Filkins LM, Zhu B, Kuchma SL, Schwartzman JD, O’Toole GA 2019 *Pseudomonas aeruginosa* can inhibit growth of *Streptococcal* species via siderophore production. J Bacteriol 201:e00014-19. doi:10.1128/JB.00014-19. 30718303 PMC6436353

[72] Dekimpe V, Deziel E 2009 Revisiting the quorum-sensing hierarchy in *Pseudomonas aeruginosa*: the transcriptional regulator RhlR regulates LasR-specific factors. Microbiology 155:712–723. doi:10.1099/mic.0.022764-0. 19246742

[73] Darch SE, Simoska O, Fitzpatrick M, Barraza JP, Stevenson KJ, Bonnecaze RT, Shear JB, Whiteley M 2018 Spatial determinants of quorum signaling in a *Pseudomonas aeruginosa* infection model. Proc Natl Acad Sci U S A 115:4779–4784. doi:10.1073/pnas.1719317115. 29666244 PMC5939081

[74] Wang M, Schaefer AL, Dandekar AA, Greenberg EP 2015 Quorum sensing and policing of *Pseudomonas aeruginosa* social cheaters. Proc Natl Acad Sci U S A 112:2187–2191. doi:10.1073/pnas.1500704112. 25646454 PMC4343120

[75] Haller S, Franchet A, Hakkim A, Chen J, Drenkard E, Yu S, Schirmeier S, Li Z, Martins N, Ausubel FM, Liegeois S, Ferrandon D 2018 Quorum-sensing regulator RhlR but not its autoinducer RhlI enables *Pseudomonas* to evade opsonization. EMBO Rep 19:e44880. doi:10.15252/embr.201744880. 29523648 PMC5934776

[76] Cruz RL, Asfahl KL, Van den Bossche S, Coenye T, Crabbé A, Dandekar AA 2020 RhlR-regulated acyl-homoserine lactone quorum sensing in a cystic fibrosis isolate of *Pseudomonas aeruginosa*. mBio 11:e00532-20. doi:10.1128/mBio.00532-20. 32265330 PMC7157775

[77] Doing G, Koeppen K, Occipinti P, Hogan D 2020 Conditional antagonism in co-cultures of *Pseudomonas aeruginosa* and *Candida albicans*: an intersection of ethanol and phosphate signaling distilled from dual-seq transcriptomics. bioRxiv doi:10.1101/2020.04.20.050765v2. PMC7480860 32813693

[78] Dandekar AA, Chugani S, Greenberg EP 2012 Bacterial quorum sensing and metabolic incentives to cooperate. Science 338:264–266. doi:10.1126/science.1227289. 23066081 PMC3587168

[79] Mund A, Diggle SP, Harrison F 2017 The fitness of *Pseudomonas aeruginosa* quorum sensing signal cheats is influenced by the diffusivity of the environment. mBio 8:e00816-17. doi:10.1128/mBio.00816-17. 28465424 PMC5414003

[80] Reid DW, Anderson GJ, Lamont IL 2009 Role of lung iron in determining the bacterial and host struggle in cystic fibrosis. Am J Physiol Lung Cell Mol Physiol 297:L795–L802. doi:10.1152/ajplung.00132.2009. 19700646

[81] Lamont IL, Konings AF, Reid DW 2009 Iron acquisition by *Pseudomonas aeruginosa* in the lungs of patients with cystic fibrosis. Biometals 22:53–60. doi:10.1007/s10534-008-9197-9. 19130260

[82] Britigan BE, Rasmussen GT, Olakanmi O, Cox CD 2000 Iron acquisition from *Pseudomonas aeruginosa* siderophores by human phagocytes: an additional mechanism of host defense through iron sequestration? Infect Immun 68:1271–1275. doi:10.1128/iai.68.3.1271-1275.2000. 10678937 PMC97278

[83] Nakashige TG, Zhang B, Krebs C, Nolan EM 2015 Human calprotectin is an iron-sequestering host-defense protein. Nat Chem Biol 11:765–771. doi:10.1038/nchembio.1891. 26302479 PMC4575267

[84] Ward PP, Conneely OM 2004 Lactoferrin: role in iron homeostasis and host defense against microbial infection. Biometals 17:203–208. doi:10.1023/b:biom.0000027693.60932.26. 15222466

[85] Michels KR, Zhang Z, Bettina AM, Cagnina RE, Stefanova D, Burdick MD, Vaulont S, Nemeth E, Ganz T, Mehrad B 2017 Hepcidin-mediated iron sequestration protects against bacterial dissemination during pneumonia. JCI Insight 2:e92002. doi:10.1172/jci.insight.92002. 28352667 PMC5358492

[86] Glasser NR, Hunter RC, Liou TG, Newman DK, Mountain West CF Consortium Investigators. 2019 Refinement of metabolite detection in cystic fibrosis sputum reveals heme correlates with lung function decline. PLoS One 14:e0226578. doi:10.1371/journal.pone.0226578. 31851705 PMC6919587

[87] Folsom JP, Parker AE, Carlson RP 2014 Physiological and proteomic analysis of *Escherichia coli* iron-limited chemostat growth. J Bacteriol 196:2748–2761. doi:10.1128/JB.01606-14. 24837288 PMC4135680

[88] Marshall B, Stintzi A, Gilmour C, Meyer JM, Poole K 2009 Citrate-mediated iron uptake in *Pseudomonas aeruginosa*: involvement of the citrate-inducible FecA receptor and the FeoB ferrous iron transporter. Microbiology 155:305–315. doi:10.1099/mic.0.023531-0. 19118371

[89] Oglesby AG, Farrow JM 3rd, Lee JH, Tomaras AP, Greenberg EP, Pesci EC, Vasil ML 2008 The influence of iron on *Pseudomonas aeruginosa* physiology: a regulatory link between iron and quorum sensing. J Biol Chem 283:15558–15567. doi:10.1074/jbc.M707840200. 18424436 PMC2414296

[90] Kim EJ, Sabra W, Zeng AP 2003 Iron deficiency leads to inhibition of oxygen transfer and enhanced formation of virulence factors in cultures of *Pseudomonas aeruginosa* PAO1. Microbiology 149:2627–2634. doi:10.1099/mic.0.26276-0. 12949186

[91] Kim EJ, Wang W, Deckwer WD, Zeng AP 2005 Expression of the quorum-sensing regulatory protein LasR is strongly affected by iron and oxygen concentrations in cultures of *Pseudomonas aeruginosa* irrespective of cell density. Microbiology 151:1127–1138. doi:10.1099/mic.0.27566-0. 15817780

[92] Cox CD 1986 Role of pyocyanin in the acquisition of iron from transferrin. Infect Immun 52:263–270. doi:10.1128/IAI.52.1.263-270.1986. 2937736 PMC262229

[93] Akintunde TA, Abioye P, Oyeleke SB, Boboye BE, Ijah UJ 2015 Remediation of iron using rhamnolipid-surfactant produced by *Pseudomonas aeruginosa*. Res J Environ Sci 9:169–177. doi:10.3923/rjes.2015.169.177.

[94] Wang S, Mulligan CN 2009 Rhamnolipid biosurfactant-enhanced soil flushing for the removal of arsenic and heavy metals from mine tailings. Process Biochem 44:296–301. doi:10.1016/j.procbio.2008.11.006.

[95] Diggle SP, Matthijs S, Wright VJ, Fletcher MP, Chhabra SR, Lamont IL, Kong X, Hider RC, Cornelis P, Camara M, Williams P 2007 The *Pseudomonas aeruginos*a 4-quinolone signal molecules HHQ and PQS play multifunctional roles in quorum sensing and iron entrapment. Chem Biol 14:87–96. doi:10.1016/j.chembiol.2006.11.014. 17254955

[96] Malhotra S, Limoli DH, English AE, Parsek MR, Wozniak DJ 2018 Mixed communities of mucoid and nonmucoid *Pseudomonas aeruginosa* exhibit enhanced resistance to host antimicrobials. mBio 9:e00275-18. doi:10.1128/mBio.00275-18. 29588399 PMC5874919

[97] Oluyombo O, Penfold CN, Diggle SP 2019 Competition in biofilms between Cystic Fibrosis isolates of *Pseudomonas aeruginosa* is shaped by R-Pyocins. mBio 10:e01828-18. doi:10.1128/mBio.01828-18. 30696740 PMC6355985

