# Supplementary figures and images for: Intraspecies Signaling between Common Variants of Pseudomonas aeruginosa Increases Production of Quorum-Sensing-Controlled Virulence Factors
Source: mBio. 2020 Aug 25;11(4):e01865-20. doi: 10.1128/mBio.01865-20 (PMC7448281; doi:10.1128/mBio.01865-20)

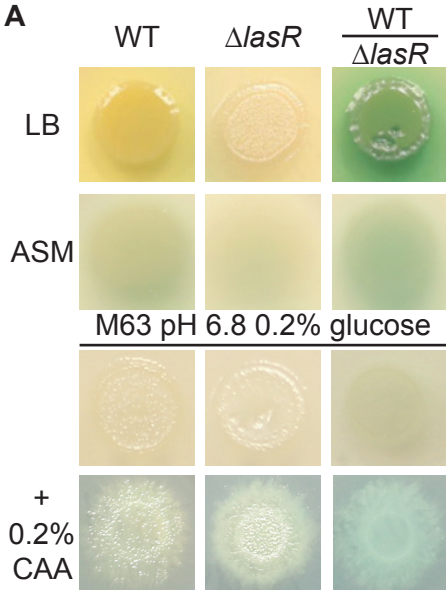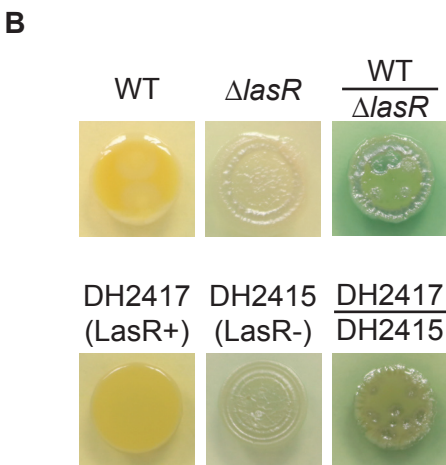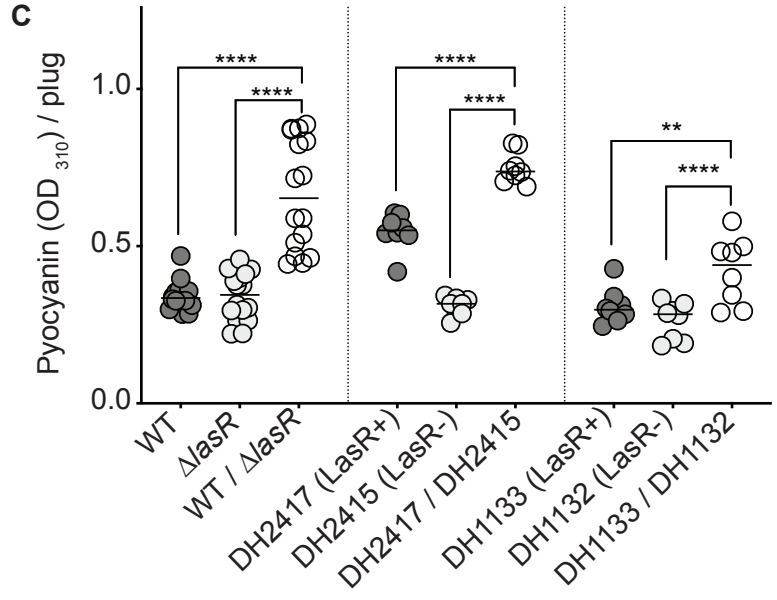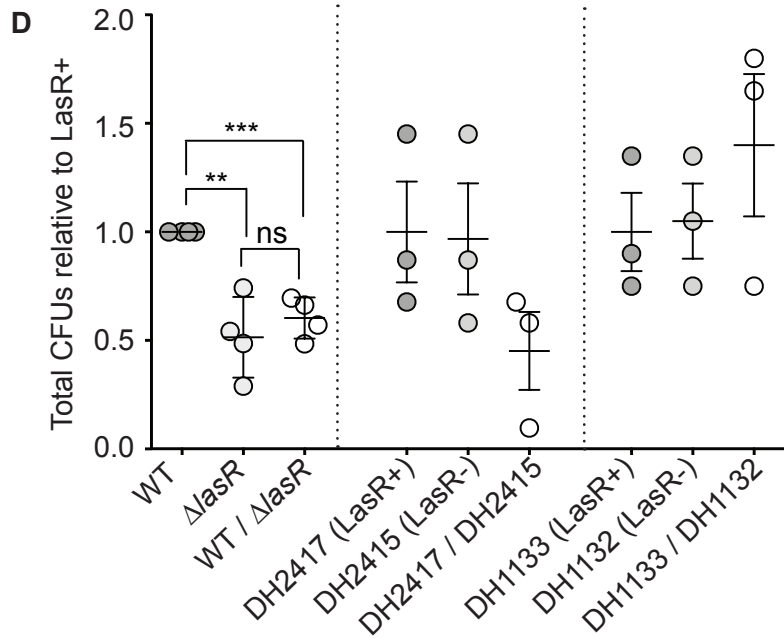

Supplement: FIG S1 [file mBio.01865-20-sf001.pdf]

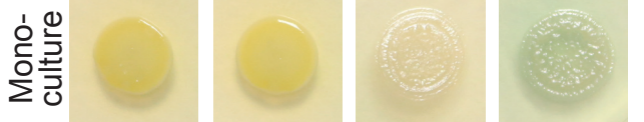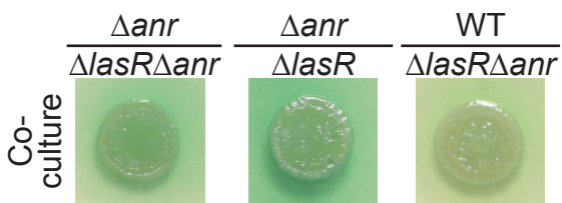

Supplement: FIG S2 [file mBio.01865-20-sf002.pdf]

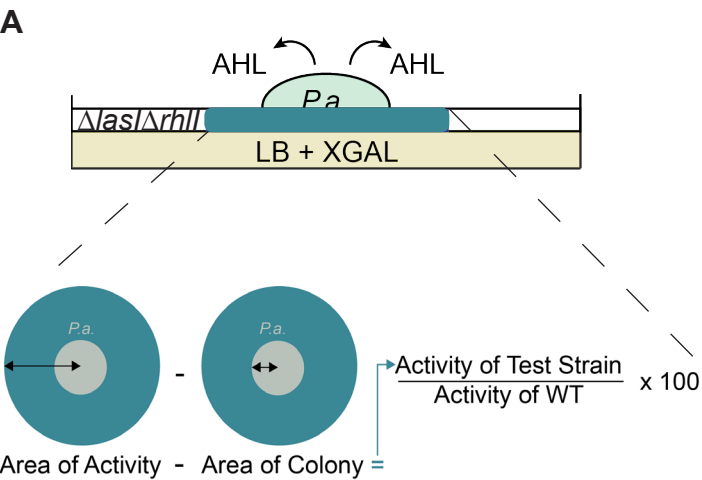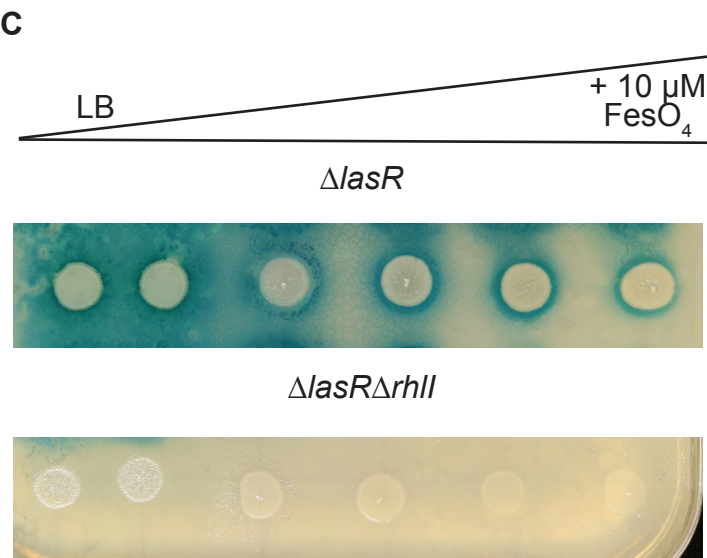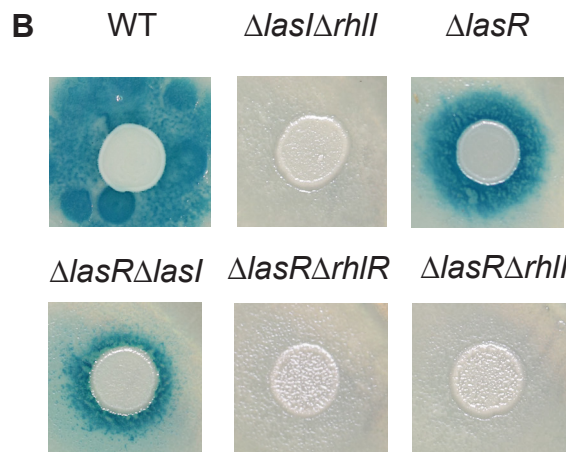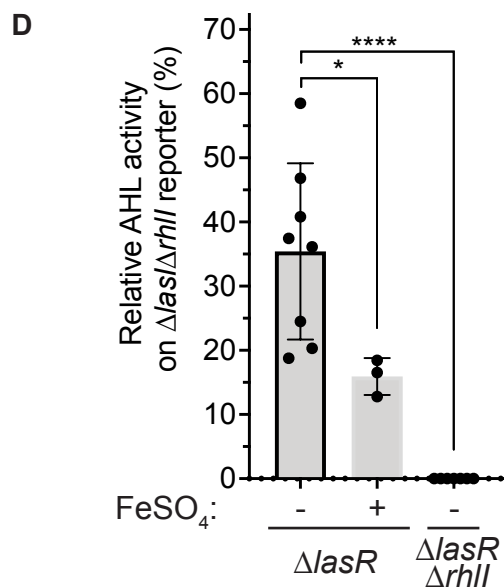

Supplement: FIG S3 [file mBio.01865-20-sf003.pdf]

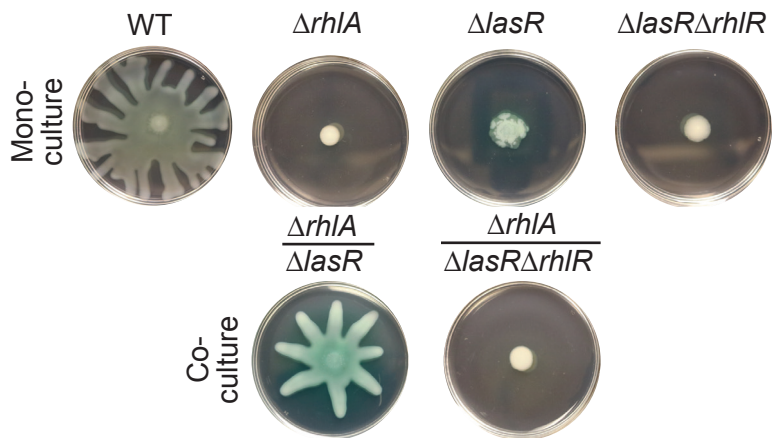

Supplement: FIG S4 [file mBio.01865-20-sf004.pdf]

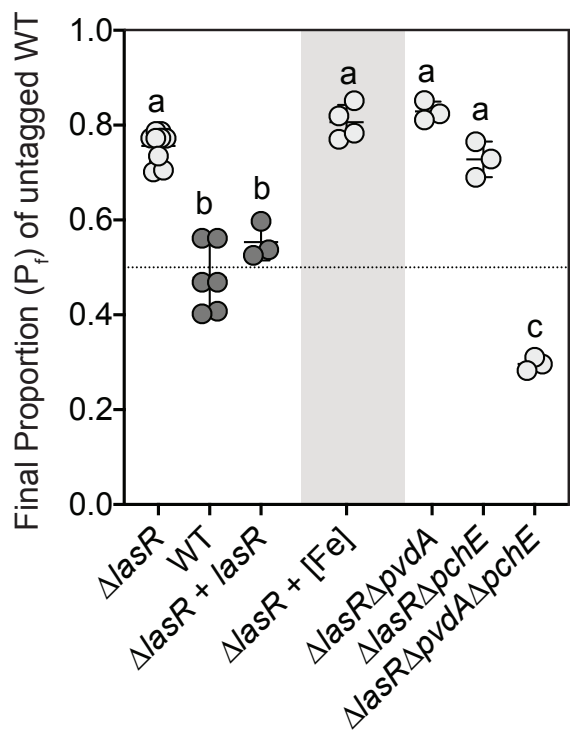

Supplement: FIG S5 [file mBio.01865-20-sf005.pdf]

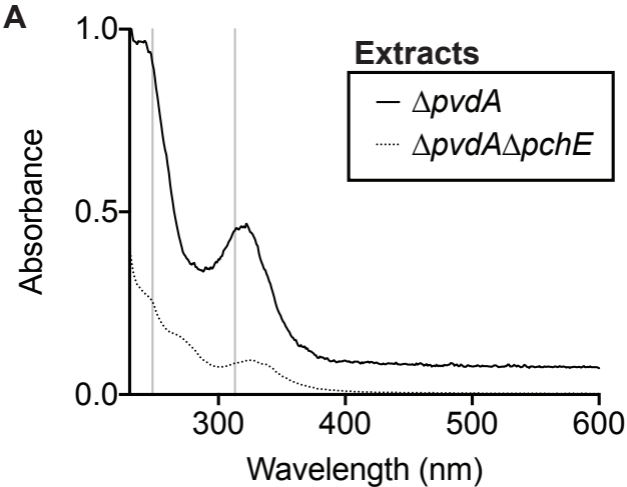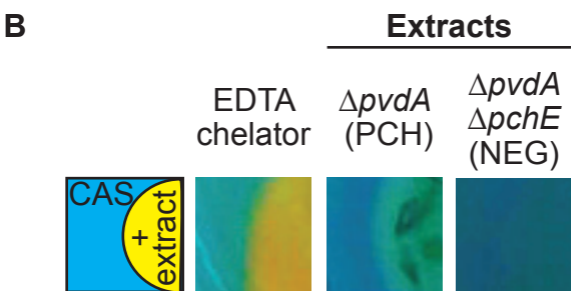

Supplement: FIG S6 [file mBio.01865-20-sf006.pdf]

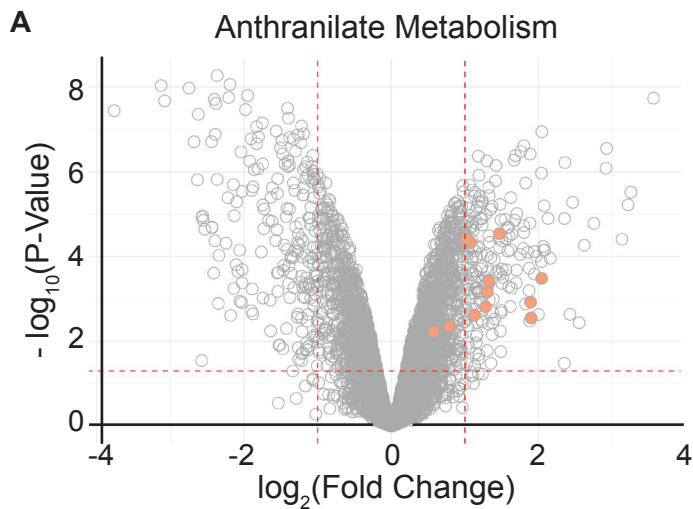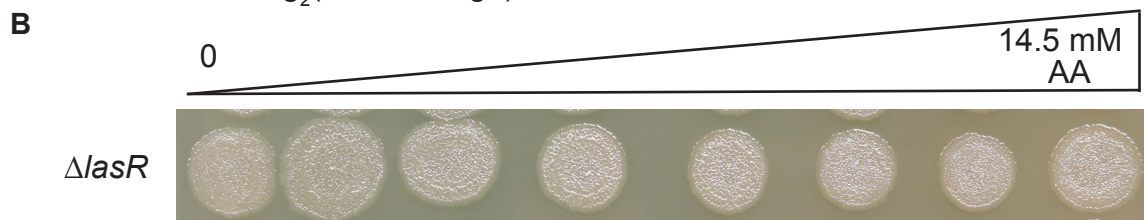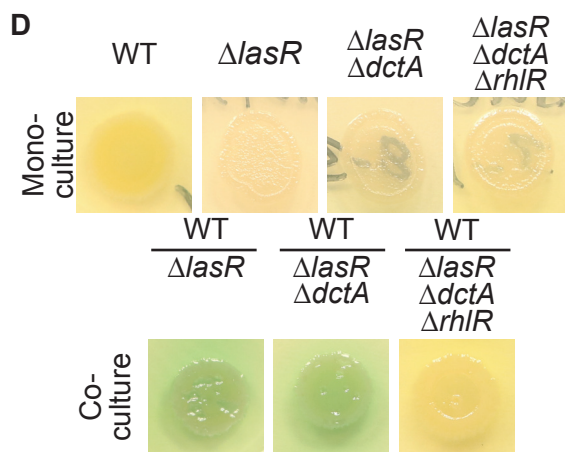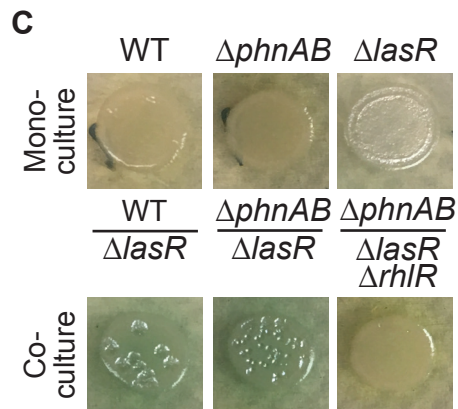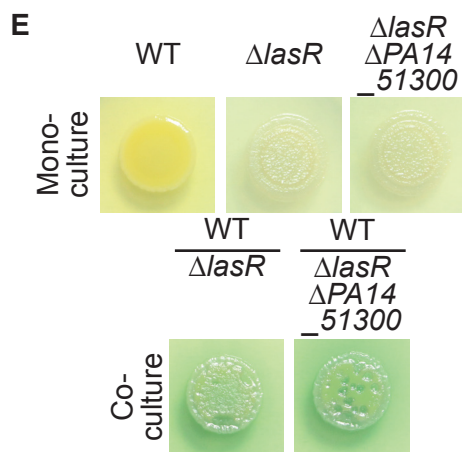

Supplement: FIG S7 [file mBio.01865-20-sf007.pdf]
