# Supplementary material for: Intraspecies Signaling between Common Variants of Pseudomonas aeruginosa Increases Production of Quorum-Sensing-Controlled Virulence Factors
Source: mBio. 2020 Aug 25;11(4):e01865-20. doi: 10.1128/mBio.01865-20 (PMC7448281; doi:10.1128/mBio.01865-20)
Supplement: TEXT S1 [file mBio.01865-20-s0001.docx]

**Supplemental Methods**

**Plasmid Construction**

Plasmid constructs for making in-frame deletions, the RhlI-HA expression (pMQ72) vector, and for *pqsA* promoter fusions were constructed using a *Saccharomyces cerevisiae* recombination technique described previously (1). The RhlI-HA expression vector with the ampicillin cassette (pMQ70) was constructed by amplifying the *rhlI* gene and HA-tag with primers including sites for cloning into pMQ70 (AmpR). All plasmids were sequenced at the Molecular Biology Core at the Geisel School of Medicine at Dartmouth. In frame-deletions and integrated promoter fusions were introduced into *P. aeruginosa* by conjugation via S17/lambda pir *E. coli*. Merodiploids were selected by drug resistance and double recombinants were obtained using sucrose counter-selection and genotype screening by PCR. Both RhlI-HA expression vectors were introduced into *P. aeruginosa* by electroporation. The ∆*lasRclpX*::Tn*M* was identified from a collection of transposon mutants in ∆*lasR* and verified by sequencing.

**Pyocyanin Quantification**

*P. aeruginosa* strains were grown in a 96-well plate containing 200 µL LB agar per well by inoculation with 5 µL of overnight cultures adjusted to OD_600 nm_ = 1. After 16 h incubation at 37 °C, two agar plugs (with indicated *P. aeruginosa* mono- or co-cultures) were added to tubes containing chloroform (500 µL), mixed by vortexing for 30 s and then centrifuged for 2 min at 13,000 RPM. The chloroform layer (200 µL) was collected into new tubes, and the chloroform extraction was repeated with an additional 500 µL of chloroform. The chloroform extracts (400 µL) were acidified with 0.2 N HCl (500 µL) and vortexed for 30 s. The pink aqueous layer containing pyocyanin was diluted 1:2 in 200 mM Tris-HCl (pH 8.0). Relative pyocyanin was measured by reading absorbance at OD_310 nm_ relative to media blank extracts. To remove background signal, the average OD_310 nm_ values of phenazine deficient ∆*lasR∆phz* were subtracted from each sample, and the values were reported per plug. Each condition had at least eight replicates each for two independent experiments.

**Colony Proximity Image analysis**

Glass beads were used to spread 50 µL of a 1:1 mixture of untagged WT and ∆*lasR* possessing the indicated promoter fusion to *lacZ* onto LB plates (2% agar) supplemented with 150 µg / mL 5-bromo-4-chloro-3-indolyl-D-galactopyranoside (X-Gal). After 16 h incubation at 37 °C, plates were placed at 4 °C for an additional 24 h to allow all ∆*lasR* colonies to develop blue coloration of various intensities. Plates were imaged on glass sheet to reduce glare using Canon EOS Rebel T6i digital camera. To process images, they were first cropped to remove background area surrounding each plate, converted to 8-bit for particle analysis in ImageJ, and the threshold was determined to count WT and ∆*lasR* CFU’s, separately. The thresholding for ∆*lasR* displayed darker blue colonies as larger areas. Colony parameters were collected for each individual colony including x, y coordinates and area for WT and ∆*lasR* colony lists. A simple distance calculation was made between every WT and ∆*lasR* colony using the x,y coordinates and the minimum distance to a WT colony was plotted for each ∆*lasR*  colony against its area value, representative of the approximate *lacZ* intensity.

**Swarming Motility Assays**

Swarm assays were performed as previously described in (2), with a few modifications. Briefly, M8 medium with 0.5 % agar was poured into 60 x 15 mm plates and allowed to dry at room temperature for 4 h prior to inoculation. LB grown cultures (16 h at 37 °C) were diluted to OD_600 nm_ = 1 in fresh LB, and co-cultures were mixed such that ∆*rhlA* was at 0.7 proportion of the final cell suspension. Each plate was inoculated with 5 µL of the final cell suspensions and incubated upright for 24 h at 37 °C in an incubated chamber followed by 12 – 16 h at room temperature. Each strain was inoculated in four replicates and assessed on at least three separate days.

**RNA Collection**

A 200 µL aliquot of optical density normalized (OD_600 nm_ = 1) cultures of PA14 or PA14 ∆*lasR* from three independent overnights were spread onto LB plates with glass beads and briefly allowed to dry. Two isopore 0.2 µm pore size PC membrane filters were stacked on the lawn (37 mm diameter filter directly on lawn then 25 mm diameter filter on top), and three 15 µL spots of normalized (OD_600 nm_ = 1) ∆*lasR* cultures were spotted on the top-most filter. After 16 h incubation at 37 °C, the top filter was collected and ∆*lasR* cells were resuspended in 1 mL LB by 5 min of vigorous shaking on the genie disruptor. Cells were pelleted for 10 min at 13,000 RPM and snap-frozen in ethanol and dry ice for RNA extraction. RNA was extracted according to manufacturer’s protocol with the QIAGEN RNeasy Mini kit and DNase treated twice with Invitrogen Turbo DNA-Free kit. DNase-treated samples were prepared for sequencing with ribodepletion and library preparation in accordance with Illumina protocols. Samples were barcoded and multiplexed in a NextSeq run by the Dartmouth Sequencing Core.

**RNA-Seq Processing**
Reads were processed using CLC Genomics Workbench wherein reads were trimmed and filtered for quality using default parameters. Reads were aligned to the *P. aeruginosa* UCPBB_PA14 genome from [www.pseudomonas.com](http://www.pseudomonas.com/). Results were exported from CLC including total counts, CPM and TPM. EdgeR was used to process differential gene expression (3). Generalized linear models with mixed effect data design matrices were used to calculate fold-change, p-value and FDR. Volcano plots and heatmaps using EdgeR output (log fold-change and -log(p-value)) were made in R (ggplot2 and pheatmap respectively) (4-6). GO term pathway enrichment analysis was carried out using PantherDB (7).

**Pyochelin extraction, quantification, and validation**

Pyochelin was extracted based on the methods of Cox et al. (8). Briefly 50 mL cultures of PA14 ∆*pvdA* and pyochelin biosynthesis deficient PA14 ∆*pvdA∆pchE* (negative control) were grown in Chelex-treated (i.e. media treated for > 2 h with 0.5 g Chelex resin per 10 mL media concentrate, followed by centrifugation and filtration with 0.22 µm pore size filter unit) LB for 16 h. Cultures were pelleted for 15 min at > 5000 RPM, and the supernatant was passed through a 0.22 µm pore size filter unit. The cell-free supernatant was acidified to pH 2 with 10 N HCl. For extraction, 5 mL ethyl acetate was added per 50 mL acidified solution in a separatory funnel. The top ethyl acetate layer was concentrated using a speedvac and quantified in 50 / 50 methanol:dH2O in a 1 mm quartz cuvette at 313 nm. Absorbance was checked from 200 - 600 nm for expected peak profile. For some extractions, a 5 µL aliquot of ∆*pvdA* extract in 50:50 methanol:dH2O was viewed under ultraviolet light for expected fluorescence relative to ∆*pvdA∆pchE* extract that dissipated upon 10 µM FeSO4 supplementation. The concentration was determined using the molar extinction coefficient at 313 nm in 50 / 50 methanol:dH2O in a 1 mm quartz cuvette (8). Upon quantification, concentrated extract was lyophilized using rotovap to yellow resin, and used within 2 days of initial extraction by resuspension in LB for supplementation. As validation of biological activity, 10 µL of extract was spotted along with Ethylenediaminetetraacetic acid (EDTA) chelator, as a control, on chrome azurol S (CAS) agar prepared as described in (9). As further evidence of biological activity, extracts containing 50 µM pyochelin were added to LB medium (alongside negative control extracts) to complement pyocyanin production of siderophore deficient ∆*pvdA∆pchE* / ∆*lasR∆pvdA∆pchE* co-cultures grown on in 12 well plate with 2 mL total volume per well. A total of 4 independent PCH+ (and 4 control) extracts were tested in 2 different complementation experiments.

**Citrate Quantification**

Citrate was quantified from cell-free supernatant of 5 mL LB-grown cultures inoculated from single colonies. Cultures were grown in quadruplicate and incubated on a roller drum for 16 h. OD _600 nm_ was recorded, and cultures were pelleted for 15 min at > 5,000 RPM. The supernatant was passed through a 0.2 µm pore size syringe filter unit. Citrate in the filtered supernatant was quantified according to manufacturer’s “manual assay” protocol (Megazyme) in ½ reactions. The extinction coefficient at OD _340 nm_ in a quartz cuvette was used to quantify concentration of citrate relative to OD _600_ at the end of the 5 min enzymatic reaction. Citrate standard and blank media conditions were included in every assay.

**Beta-galactosidase (β-Gal) assays**

Cells with a promoter fusion to *lacZ - GFP* integrated at the *att* locus were grown in 5 mL cultures of LB at 37°C for 16 h. The cultures were diluted to a starting OD _600 nm_ of 1 and 5 µL were spotted onto LB agar plates $\pm$ 20 mM pH 7 citrate in triplicate. After 24 h at 37 °C, colony biofilms were cored, resuspended in 500 µL LB by vigorous shaking on the Genie Disrupter for 5 min as previously described, and β-Gal activity was measured as described by Miller (10). The average for each experiment was reported across 3 - 4 independent days.

**Western Blot**

Strains were grown in LB broth under selection (60 μg / mL gentamycin or 60 μg / mL carbenicillin as appropriate) for 16 h at 37 °C on a roller drum, and 5 μL of culture were spotted onto LB plates under selection with 0.2% L-arabinose (v/v) and +/- 20 mM indicated carbon source. Inoculated plates were incubated at 37 °C for 16 h. Colony biofilms were resuspended in 325 μL of Laemmli buffer without reducing agent and heated at 100 °C for 15 min. Protein was quantified on 1:10 dilution of protein sample according to standard procedure via Thermo scientific BCA Protein Assay Kit. Reducing agent was added and samples were run on a 4 - 15% SDS gradient gel (Bio-Rad) at 60 V for 40 min followed by 110 V for 45 min. After SDS page electrophoresis, protein was transferred onto LF-PVDF membrane (Bio-Rad) using the mixed molecular weight option on a turbo blot apparatus (Bio-Rad). After transfer, the membrane was dried, rehydrated, and then a total protein stain was run according to manufacturer’s procedure (Li-Cor). Following protein quantification, the membrane was incubated in TBS blocking buffer (Li-Cor) for 1 h, and then purified anti-HA mouse monoclonal antibody (Biolegend) in TBS blocking buffer (1:2,500 dilution) for 1 hr. Following primary antibody detection, the membrane was washed 4 times in TBST 0.1%. Secondary detection was done by incubation with goat anti-mouse in TBS blocking buffer (1:15000 dilution) for 1 hour in the dark. Following detection, the membrane was washed 3 times in TBST 0.1% and once in TBS. The membrane was then dried and imaged on the Li-Cor Odyssey CLx imager relative to REVERT total protein stain.

**Acyl-homoserine lactone (AHL) autoinducer Activity Assay**

The AHL deficient PAO1 ∆*lasI∆rhlI* containing an AHL responsive promoter to *lacZ* (qsc131) (11) was grown in LB (16 h 37 °C) and normalized to OD_600 nm_  = 0.1. A 200 µL aliquot of the AHL-responsive ∆*lasI∆rhlI* reporter was spread with sterile glass beads onto LB plates containing 150 µg / mL X-Gal (and 10 µM FeSO_4_ when indicated), and briefly allowed to dry. Test strains were grown in LB, normalized to OD_600 nm_  = 1, and a 5 µL aliquot of normalized culture was spotted on top of the reporter lawn. The described co-cultures were incubated at 37 °C for 16 h. The relative AHL activity was quantified by subtracting the area of the colony from the area of the blue circle of activity, and values were presented as percentages of WT activity in that experiment. Data were collected on at least 3 independent days.

1. Shanks RM, Caiazza NC, Hinsa SM, Toutain CM, O'Toole GA. 2006. *Saccharomyces cerevisiae*-based molecular tool kit for manipulation of genes from gram-negative bacteria. Appl Environ Microbiol 72:5027-36.

2. Ha DG, Richman ME, O'Toole GA. 2014. Deletion mutant library for investigation of functional outputs of cyclic diguanylate metabolism in *Pseudomonas aeruginosa* PA14. Appl Environ Microbiol 80:3384-93.

3. Robinson M, McCarthy D, Smyth G. 2010. edgeR: a Bioconductor package for differential expression analysis of digital gene expression data. Bioinformatics 26:139-140.

4. Wickham H. 2016. ggplot2: Elegent Graphics for Data Analysis, Springer-Verlag, New York. <https://ggplot2.tidyverse.org>.

5. Team RDC. 2010. R: A language and environment for statistical computing, R Foundation for Statistical Computing, Vienna, Austria. <http://www.R-project.org>.

6. Kolde R. 2019. pheatmap: Pretty Heatmaps, <https://CRAN.R-project.org/package=pheatmap>.

7. Mi H, Muruganujan A, Ebert D, Huang X, Thomas PD. 2018. PANTHER version 14: more genomes, a new PANTHER GO-slim and improvements in enrichment analysis tools. Nucleic Acids Res 47:D419-D426.

8. Ankenbauer RG, Toyokuni T, Staley A, Rinehart KL, Cox CD. 1988. Synthesis and biological activity of pyochelin, a siderophore of *Pseudomonas aeruginosa*. J Bacteriol 170:5344-5351.

9. Louden BC, Haarmann D, Lynne AM. 2011. Use of blue agar CAS assay for siderophore detection. J Microbiol Biol Educ 12:51-3.

10. Miller JH. 1992. A Short Course in Bacterial Genetics. Cold Spring Harbor Press.

11. Whiteley M, Lee KM, Greenberg EP. 1999. Identification of genes controlled by quorum sensing in *Pseudomonas aeruginosa*. Proc Natl Acad Sci U S A 96:13904-9.
